# Controlled release of H_2_S and NO gases through CO_2_-stimulated anion exchange

**DOI:** 10.1038/s41467-019-14270-3

**Published:** 2020-01-23

**Authors:** Shinsuke Ishihara, Nobuo Iyi

**Affiliations:** International Center for Materials Nanoarchitectonics (WPI-MANA), National Institute for Materials Science (NIMS), 1-1 Namiki, Tsukuba, Ibaraki 305-0044 Japan

**Keywords:** Chemistry, Materials science

## Abstract

Difficulties related to handling gases are a common bottleneck for applications. Although solid materials that release gas molecules under external stimuli exist, they require an external energy or a device for reliable operation. Herein, we report a CO_2_ stimulus for controlled release of p.p.m.-level functional gases from solid materials. A CO_2_-preferential anion-exchange property of layered double hydroxides and redox reactions in gas molecules are combined to release various gases (including H_2_S and NO) under ambient air from HS^−^ and NO_2_^−^-incorporated layered double hydroxides, respectively. The profiles of gas release are mainly governed by the difference of p*K*_a_ between H_2_CO_3_ and resulting acids (formed through protonation of interlayer anions), and are not so susceptible to the variation of relative humidity in air. Moreover, structural modulation of solid materials enables fine control of the gas release profiles. The use of safe, ubiquitous, and nearly constant (~400 p.p.m. in atmosphere) CO_2_ stimulus offers broad applications for functional gases.

## Abstract (web summary)

Solid materials releasing gas molecules under external stimuli usually require external energy for reliable operation. Here the authors synthesize layered double hydroxides for the autonomous release of H_2_S and NO gases in response to CO_2_-stimulus.

## Introduction

Gas molecules play key roles in many research fields and their practical utility depends on the safety and cost-effectivity of the gas delivery system (particularly when gases are toxic, labile, or flammable)^1–3^. The use of high-pressure gas cylinders is often the source of safety concerns; therefore, solid materials that release gas molecules under external stimuli (e.g., heat, light, and vacuum) have attracted particular interest^4–6^. However, such conventional stimuli require external energy (e.g., electricity) and controlling device for reliable operation. By contrast, the use of aerial components as stimuli for gas release is attractive in that air is safe, charge-free, and available anytime anywhere. Some zeolites and metal organic frameworks that capture and release gases have been reported^7–9^. In particular, controlled release of H_2_S and NO is valuable for medical application, because these gases are physiologically active (i.e., they exhibit effects such as anti-inflammatory, anti-oxidative, cytoprotective, and vasodilatory effects) at p.p.m.-level concentrations but are toxic at higher concentrations and labile under air^10–12^. These solids bind gases at coordinatively unsaturated transition metal cation sites and release them under moist air through the replacement of gas molecules with water. However, the water-triggered gas-release system could be susceptible to the variation of relative humidity (RH) and long-term release of gases were, in most cases, demonstrated under fixed and low RH condition (10~20%RH)^7,8^. In addition, initial burst release of highly concentrated gases tends to be observed.

**Fig. 1:**
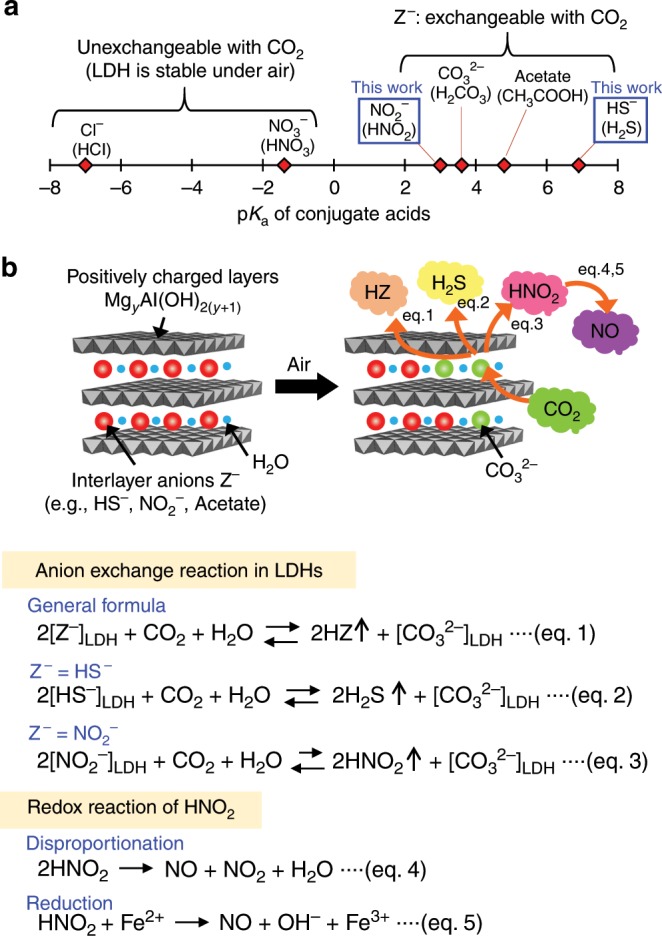
Design of solid materials that release functional gases in response to CO_2_. **a** Susceptibility of interlayer anions (Cl^−^, NO_3_^−^, NO_2_^−^, CO_3_^2−^, acetate, and HS^−^) of LDHs for CO_2_-stimulated anion exchange, showing correlation with p*K*_a_ values of the conjugate acids. Z^−^ denotes a conjugate base of volatile weak acid. **b** Release of various gases (HZ, H_2_S, HNO_2_, or NO) from LDHs through anion exchange between interlayer anions (Z^−^, HS^−^, or NO_2_^−^) and aerial CO_2_ (eq. 1–3), disproportionation (eq. 4), and reduction (eq. 5). Note that interlayer- and aerial-H_2_O are available in eq. 1–3.

Layered double hydroxides (LDHs) are inorganic layered materials with the general formula M^II^_*y*_M^III^(OH)_2(*y*+1)_(X^n−^)_1/n_·*m*H_2_O, where M^II^, M^III^, X^n−^, and *m*H_2_O are, respectively, a divalent metal cation (*y* is in the range of 2−4), a trivalent metal cation, an *n*-valent anion, and hydrated water (*m* depends on the humidity of the environment) (Fig. 1)^13^. M^II^_*y*_M^III^(OH)_2(*y*+1)_ forms a positively charged two-dimensional (2D) layer and both charge-compensating anion (X^n−^) and hydrated water (*m*H_2_O) are located within the interlayer. Previously, we reported that some conjugate anions of weak acids (e.g., acetate and carbonate) in the interlayer of Mg/Al-type LDHs tend to get exchanged with CO_3_^2−^ derived from aerial CO_2_^14–16^. The CO_2_-stimulated anion-exchange phenomenon at the air–solid interface inspired us to explore a novel class of gas-releasing materials.

Herein, we report solid materials that autonomously release functional gases in response to CO_2_ stimulus. Anions of weak acids (Z^−^; p*K*_a_ > ~2) involved in the interlayer of Mg/Al-type LDHs can be protonated by H_2_CO_3_ (p*K*_a_ = 3.6) derived from aerial CO_2_ and interlayer H_2_O, leading to autonomous release of protic gases (HZ) (Fig. 1b, eq. 1). Interlayer HS^−^ or NO_2_^−^ in LDHs is protonated with aerial CO_2_ and H_2_O, resulting in the autonomous release of H_2_S or HNO_2_ under air (Fig. 1b, eq. 2 and 3). HNO_2_ is convertible to NO through an automatic disproportionation reaction or subsequent treatment with a reducing agent (Fig. 1b, eq. 4 and 5)^17^. Although there are some precedent reports of generating NO from NO_2_^−^ through chemical reaction in solution^18–22^, NO_2_^−^-incorporated LDHs offer all-solid system for NO release. Profiles (concentration and duration) of gas release are basically dependent on the p*K*_a_ of the resulting acids, because the protonation equilibrium between interlayer anions and H_2_CO_3_ (from CO_2_ and H_2_O) governs the chemical events. We also demonstrate that profiles of gas release can be controlled by various factors such as chemical composition of LDHs, diffusion of gases and ions, and chemical equilibrium. The profiles of gas release from LDH are not so susceptible to the variation of RH due to abundant water inherently present in the interlayer. This could be advantageous for release of functional gases under ambient air, because RH is quite variable from moment to moment. In contrast, concentration of CO_2_ in ambient air is almost constant (around ~400 p.p.m.), offering controlled release of functional gases without precise adjustment of conditions for gas release. Moreover, Mg/Al-type LDHs is transition-metal-free and biocompatible^23^. Thus, our low-cost and safe-to-handle materials are feasible for creating a disposable medical system for a controlled release of p.p.m.-level physiologically active gases under ambient air.

This work reveals that LDH is an attractive material for gas release and the CO_2_-driven system is potentially useful for expanding opportunities of utilizing functional gases in society.

## Results and discussion

### Synthesis and characterization of H_2_S-releasing LDHs

**Fig. 2:**
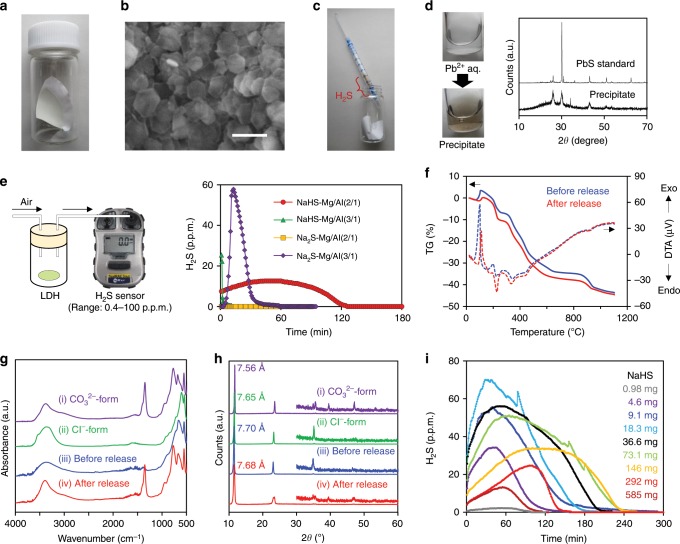
Characterization of H_2_S-releasing layered double hydroxides. **a** Photograph of NaHS-Mg/Al(2/1) embedded on membrane filter (stored in the glass vial purged with dry N_2_). **b** SEM image of NaHS-Mg/Al(2/1) after H_2_S release (scale bar: 2 μm). **c** H_2_S release from NaHS-Mg/Al(2/1) confirmed by detector tube. **d** Powder XRD pattern of black precipitate obtained by reaction of aqueous Pb^2+^ with released gas. **e** Continuous monitoring of H_2_S released from LDHs under standard flow condition; 0.020 mmol of LDHs (5.0 mg of Mg/Al(2/1) and 6.25 mg of Mg/Al(3/1)) were tested. Thermogravimetry-differential thermal analysis (TG-DTA) profiles (**f**), IR spectra (**g**), and powder XRD patterns (**h**) of NaHS-Mg/Al(2/1) before and after H_2_S release. For comparison, IR and XRD data of starting materials are also shown. **i** Optimizing amount of NaHS∙*n*H_2_O used in preparing NaHS-Mg/Al(2/1) from 40 mg of Cl^−^-type LDH; 20 mg of LDH was tested under standard flow condition.

LDHs involving HS^−^ or S^2−^, for H_2_S release, were synthesized by two-step anion-exchange reactions^24^. As starting materials, CO_3_^2−^-type LDHs with Mg:Al ratio of 3:1 (Mg_3_Al(OH)_8_(CO_3_^2−^)_0.5_·2H_2_O) and 2:1 (Mg_2_Al(OH)_6_(CO_3_^2−^)_0.5_·2H_2_O) were utilized. The former is commercially available and the latter was synthesized by a hydrothermal reaction^25^. As CO_3_^2−^ of LDH is hardly exchangeable with other anions under normal anion-exchange conditions, CO_3_^2−^ in LDH was first replaced with Cl^−^ using reported de-intercalation method^26^. Then, the resulting Cl^−^-type LDHs (Mg:Al = 2:1 or 3:1) dispersed in degassed deionized water were reacted with 10 equivalent (in mole) of NaHS∙*n*H_2_O or Na_2_S∙9H_2_O for 2 days under N_2_, as summarized in Supplementary Table 1. Solid materials were collected by filtration, washed with degassed deionized water, and then dried in vacuum (all performed under N_2_ atmosphere) to afford four types of products (NaHS-Mg/Al(2/1), NaHS-Mg/Al(3/1), Na_2_S-Mg/Al(2/1), and Na_2_S-Mg/Al(3/1); see Fig. 2a for a typical image). The products were preserved in a sealed pack for isolation from air (Supplementary Fig. 9). As far as we know, HS^−^- or S^2−^-incorporated LDHs produced in an inert atmosphere throughout syntheses and preservation have not been reported. Although there are some reports that claim the synthesis of HS^−^- or S^2−^-incorporated LDHs, they were synthesized and/or preserved under air^27,28^. As these materials are sensitive to air, their products produced without care about atmosphere must be different from ours. A scanning electron microscopy (SEM) image showed that the morphology (hexagonal plate) of LDH was maintained after multi-step anion-exchange reactions (Fig. 2b).

NaHS-Mg/Al(2/1) released odor characteristic to H_2_S for over 1 h when exposed to air [CAUTION!!] and the response of the detector tube was positive (Fig. 2c). In addition, the released gas was confirmed as H_2_S through the formation of PbS upon interaction with Pb^2+^ (Fig. 2d). Moreover, SO_2_ was not detected (<0.01 p.p.m.) in ~10 p.p.m. H_2_S by the detector tube. The concentration of H_2_S released from LDHs was continuously monitored by an electrochemical sensor under the standard flow conditions employed in this study (air, 50%RH, 100 mL min^−1^, 20 °C) and NaHS-Mg/Al(2/1) demonstrated a release of ~10 p.p.m. H_2_S for 2 h (Fig. 2e). On the other hand, Na_2_S-Mg/Al(2/1) did not release H_2_S. NaHS-Mg/Al(3/1) and Na_2_S-Mg/Al(3/1) released concentrated (over 25 p.p.m.) H_2_S, but the release duration was not as long as that of NaHS-Mg/Al(2/1). A release profile of NaHS-Mg/Al(2/1) can be explained by a narrow interlayer distance of Mg/Al = 2/1-type LDHs^16^, which suppresses the interaction between interlayer anions and aerial components.

Thermogravimetry-differential thermal analysis showed that NaHS-Mg/Al(2/1) involves HS^−^, which demonstrate exothermal oxidation into S_2_O_3_^2−^ at around 65–100 °C in air (Fig. 2f)^27,28^. In contrast, exothermal signals were not observed for Na_2_S-Mg/Al(2/1) at 65–100 °C, indicating that sulfur sources were not incorporated (Supplementary Fig. 10). After H_2_S release ceased, Fourier-transform infrared (IR) and powder X-ray diffraction (XRD) analyses of NaHS-Mg/Al(2/1) indicated that HS^−^ was partly replaced with CO_3_^2−^(Fig. 2g, h), supporting the CO_2_-stimulated anion-exchange mechanism. After H_2_S release, only a trace amount of S_2_O_3_^2−^ (1000–1200 cm^−1^) was observed in the IR spectrum of NaHS-Mg/Al(2/1). In contrast, NaHS-Mg/Al(3/1), which ceased H_2_S release within a few minutes (Fig. 2e), showed an intense IR signal from S_2_O_3_^2−^ due to HS^−^ oxidation (Supplementary Fig. 11)^28^. Thus, HS^−^ tolerance against aerial oxidation is a crucial factor for long-term release of H_2_S.

The amount of NaHS∙*n*H_2_O used in the synthesis of NaHS-Mg/Al(2/1)-type LDH was varied to find the optimum condition for obtaining the largest H_2_S release (Fig. 2i). The concentration of the released H_2_S increased with an increase in the amount of NaHS∙*n*H_2_O from 0.98 mg to 18.3 mg for 40 mg Cl^−^-type LDH and the gross release reached maximum at 36.6 mg NaHS∙*n*H_2_O (=2.6 equivalent in mole for Cl^−^). However, further addition of NaHS∙*n*H_2_O would reduce the gross release of H_2_S, presumably due to increased basicity (i.e., OH^−^) and impurities involved in the reaction solution.

Assuming that the chemical composition of NaHS-Mg/Al(2/1) with a maximum gross release is Mg_2_Al(OH)_6_(HS^−^)·2H_2_O (Mw = 246.8 g mol^−1^), 20 mg of the product involves 81 μmol of HS^−^, which corresponds to a release of 76 p.p.m. H_2_S for 240 min (under 100 mL min^−1^). The actual amount of H_2_S released from NaHS-Mg/Al(2/1) was about half the expected value (Fig. 2i), which is attributable to an incomplete anion exchange from Cl^−^ to HS^−^ (see Supplementary Discussion and Supplementary Fig. 21 for discussion on chemical formula of NaHS-Mg/Al(2/1)).

It is widely known that NaHS and Na_2_S also release odor characteristic to H_2_S and we confirmed that these simple salts release H_2_S under air (Supplementary Fig. 12). H_2_S has rather high p*K*_a_ so that even simple salts can participate in CO_2_-stimulated protonation equilibrium. However, these salts are deliquescent and strongly basic (pH ≥ 12). In contrast, NaHS-Mg/Al(2/1) is non-deliquescent, insoluble in water, and nearly neutral (pH ≈ 8), even when wet with water (Supplementary Fig. 12). Moreover, LDHs offer fine control of H_2_S release by tuning of their chemical compositions. Mg/Al-type LDH, known as hydrotalcite, is toxic-heavy-metal-free, biocompatible, and practically utilized as an antacid drug^23^. These features of LDHs are advantageous for medical applications.

We note a fundamental importance of LDH in gas release. Our previous study^15^ revealed that interlayer CO_3_^2−^ of LDH is dynamically exchanging with aerial CO_2_, although CO_3_^2−^ of a simple salt (^13^C-labeled Na_2_CO_3_) did not exchange. The result implies that interlayer anions in LDH is activated to CO_2_-stimulus due to 2D structure and high affinity of LDH to CO_2_ (while contact of interlayer anions and aerial components is appropriately regulated). The concept is generally applicable and will guide a design of controlled release of various types of gases.

### Controlled release of H_2_S from LDHs

**Fig. 3:**
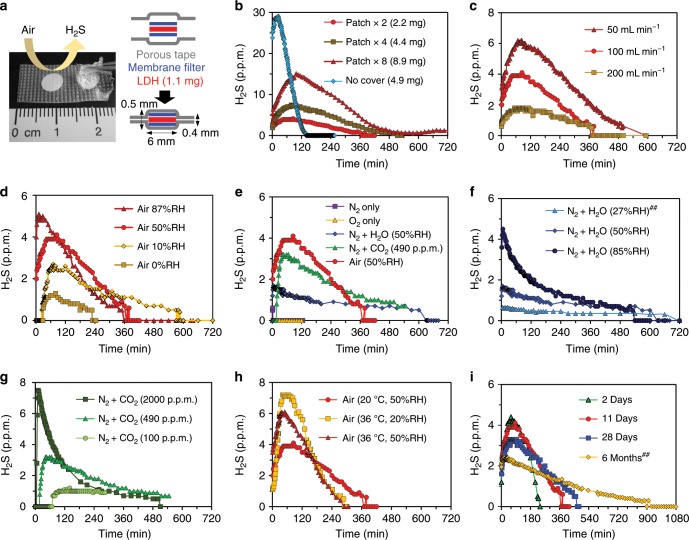
H_2_S release profiles from NaHS-Mg/Al(2/1) sandwiched between porous tapes. **a** Photograph and illustration of H_2_S release patches. One patch contains 1.1 mg of LDH. **b**–**i** Influences of various factor for H_2_S release profiles^#^; **b** patch numbers (release from noncovered sample is also shown for comparison), **c** flow rate of air, **d** humidity in air, **e** type of carrier gases, **f** humidity in N_2_, **g** CO_2_ concentration in dry N_2_, **h** effect of temperature and humidity, and **i** aging effect. ^#^Unless noted, H_2_S release from two patches was tested under the standard flow condition. ^##^Release from four patches was halved. See Supplementary Figs. 1 and 2 for experimental set-up.

To reduce the interaction between NaHS-Mg/Al(2/1) and air for longer H_2_S release, assembled materials with LDHs wrapped with porous tapes were prepared. About 1.1 mg of NaHS-Mg/Al(2/1), which was synthesized under the optimum conditions using 40 mg LDH and 36.6 mg NaHS∙*n*H_2_O (Fig. 2i), was sandwiched between the membrane filters and further between the porous tapes (Fig. 3a and see Supplementary Fig. 13 for details). As a result, the H_2_S release profile was significantly improved compared to that of the bare (i.e., noncovered) material (Fig. 3b). In addition, this patch-like assembly was effective in holding the LDH powder.

The H_2_S release profiles of the patch-like assembly were investigated under various conditions to probe the mechanisms and controllability of H_2_S release. The H_2_S concentration was almost proportional to the number of patches (Fig. 3b), which indicates ease of control of the gas concentration. The flow rate also affects the measured concentration: the H_2_S concentration was inversely proportional to the flow rate of air (Fig. 3c). This means that the quantity of H_2_S released from LDHs was almost constant. The RH in air did not affect so much on the concentration of H_2_S (under practical range; 10~87%RH), although the concentration reduced for several times under fully dry air (Fig. 3d). H_2_S release was not observed under dry N_2_ and O_2_ (Fig. 3e), but addition of humidity to N_2_ induced H_2_S release (Fig. 3f) due to the presence of equilibrium between interlayer HS^−^ and H_2_O (i.e., [HS^−^]_LDH_ + H_2_O → H_2_S↑ + [OH^−^]_LDH_). Addition of CO_2_ into dry N_2_ also caused H_2_S release (Fig. 3g) and it is clear that the gas release from LDHs is strongly dependent on CO_2_ concentration. However, CO_2_ concentration in air is almost constant (~400 p.p.m.), which is in contrast to RH that can change dramatically from moment to moment. In this process, the proton source must be interlayer H_2_O and the overall reaction is expressed as 2[HS^−^]_LDH_ + CO_2_ + [H_2_O]_LDH_ → 2H_2_S↑ + [CO_3_^2−^]_LDH_. The activity of H_2_S release was gradually quenched when exposed to dry O_2_ beforehand, which is attributable to HS^−^ oxidation (Supplementary Fig. 14). The H_2_S concentration was slightly increased when heated to 36 °C (Fig. 3h), presumably due to accelerated diffusion of gas molecules and anions within the interlayer. LDHs preserved in a sealed pack for six months demonstrated a rather flat and elongated H_2_S release (Fig. 3i). Mechanism of the aging effect could be homogenized distribution of HS^−^ within the interlayer. In fact, the aging effect can be accelerated by thermal treatment (e.g., 60 °C for several days) (see Supplementary Discussion and Supplementary Fig. 15 and 23 for discussion on the aging effect).

### Controlled release of NO from LDHs

**Fig. 4:**
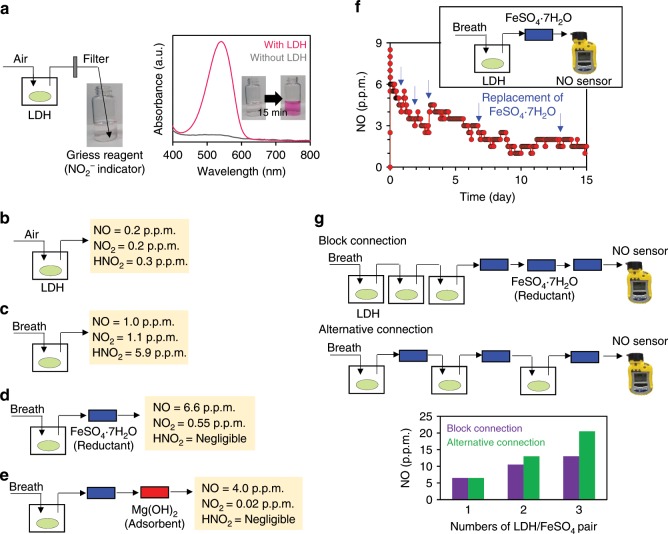
Release of HNO_2_ and conversion to NO. **a** Color change of Griess reagent showing the presence of HNO_2_. **b**–**e** Concentrations of NO, NO_2_, and HNO_2_ released from 100 mg NaNO_2_-Mg/Al(3/1) (determined by detector tube). See Supplementary Fig. 3 for experimental details. Air (20 °C, 35%RH) or exhaled breath were applied at flow rate of 100 mL min^−1^. Released gases were passed through FeSO_4_∙7H_2_O and Mg(OH)_2_ loaded in the glass tube. **f** NO-release profile from 100 mg NaNO_2_-Mg/Al(3/1) under exhaled-breath flow (50 mL min^−1^). FeSO_4_∙7H_2_O was occasionally replaced with new ones (indicated by blue arrow). See Supplementary Fig. 4 for experimental details. **g** Tandemly connected NaNO_2_-Mg/Al(3/1) and FeSO_4_∙7H_2_O (in block or alternative manner) for accumulating the concentration of NO under exhaled-breath flow (50 mL min^−1^). Each vial contains 100 mg NaNO_2_-Mg/Al(3/1). The NO concentration is monitored after stabilization for 15 min.

LDHs involving NO_2_^−^ were synthesized from Cl^−^-type LDHs (Mg:Al = 2:1 or 3:1) and NaNO_2_, yielding NaNO_2_-Mg/Al(2/1) and NaNO_2_-Mg/Al(3/1) (183 mg NaNO_2_ used for 40 mg LDHs; see Supplementary Method, Discussion, and Fig. 22 for optimization of mixing ratio and discussion on chemical formula of the product). Release of HNO_2_ from NaNO_2_-Mg/Al(3/1) under air is suggested by visible color change of Griess reagent, which is a NO_2_^−^ indicator^29^ (Fig. 4a). The detector tube for NO + NO_2_ demonstrates a positive response (0.7 p.p.m.) for gases released from 100 mg NaNO_2_-Mg/Al(3/1) under air (Fig. 4b). It is noteworthy that the detector tube for NO + NO_2_ (“Tube-A”) was equipped with the strong oxidant (Cr^6+^ + H_2_SO_4_) part at the entry for conversion of NO to NO_2_ and its response should also involve the contribution of HNO_2_. Thus, the total concentration, NO + NO_2_ + HNO_2_, was 0.7 p.p.m. The concentration of nitrogenous gases released from LDHs was two to three orders of magnitude lower than that of H_2_S, because H_2_CO_3_ formed by CO_2_ and water can afford fewer protons to NO_2_^−^ than that to HS^−^ due to lower p*K*_*a*_ of HNO_2_ ( = ~3.0)^30^. On the other hand, release of nitrogenous gases was much longer, and continued for at least one day, maintaining similar concentration. Besides, NO_2_ measured by another type of detector tube without the oxidant part (“Tube-B”) was 0.2 p.p.m. NO was measured by combining two detector tubes, as follows. First, NO_2_ and HNO_2_ were removed by detector tube “B” for NO_2_, which contains *o*-tolidine (aromatic amine) as an indicator. Here, acidic HNO_2_ was removed by passing through this tube, but did not change color of the tube. In fact, after this treatment, the released gas did not change color of the Griess reagent, indicating complete removal of HNO_2_ together with NO_2_. Then, NO was determined as 0.2 p.p.m. by the next detector tube, “A” for NO + NO_2_. Thus, HNO_2_ was estimated to be 0.3 p.p.m. by subtracting 0.2 p.p.m. of NO_2_ and 0.2 p.p.m. of NO from the total 0.7 p.p.m. The release of equal amounts (0.2 p.p.m.) of NO and NO_2_ indicates that these gases are derived from the disproportionation of HNO_2_ (eq. 4 in Fig. 1b). Release of nitrogenous gases (NO + NO_2_ + HNO_2_) under air flow (100 mL min^−1^) increased a little with humidity (0.5 p.p.m. at 1.8%RH, 0.7 p.p.m. at 35%RH, 1.1 p.p.m. at 82%RH, and 1.6 p.p.m. at 90%RH), but maintained similar order of concentration under practical RH (i.e., 35~90%RH).

Compared with NaNO_2_-Mg/Al(3/1), release of nitrogenous gases from NaNO_2_-Mg/Al(2/1) was considerably small in air (~0.1 p.p.m. or less) in the initial 2 h, whereas that of gases was gradually increased to ~1.5 p.p.m. when the sample was further left in air. This delayed release profile resembles the case of NaHS-Mg/Al(2/1), where the anion-exchange reaction was regulated due to the narrow interlayer space.

Exhaled breath (4.0% CO_2_, nearly saturated humidity, 100 mL min^−1^) was applied to 100 mg NaNO_2_-Mg/Al(3/1) for promoting protonation of interlayer NO_2_^−^ (eq. 3 in Fig. 1b) and NO, NO_2_, and HNO_2_ measured by detector tubes were 1.0, 1.1, and 5.9 p.p.m., respectively (Fig. 4c). HNO_2_ can be reduced to NO using Fe^2+^ (eq. 5 in Fig. 1b)^17^, and insertion of the FeSO_4_∙7H_2_O column into the flow line successfully increased NO concentration to 6.6 p.p.m. (Fig. 4d). NO_2_ was decreased to 0.55 p.p.m., presumably due to partial reduction to NO and/or adsorption on FeSO_4_∙7H_2_O. The total amount of nitrogenous gases (NO + NO_2_ + HNO_2_) was ~7.0 p.p.m., which means that the unreacted HNO_2_ was negligible. The remaining NO_2_ could be removed down to 0.02 p.p.m. (only twice of the atmospheric level) using Mg(OH)_2_, a selective adsorbent for acidic gases (Fig. 4e)^31,32^.

Release of NO from NaNO_2_-Mg/Al(3/1) under exhaled breath continued over 2 weeks, as monitored by an electrochemical NO sensor, and its half-life of release was ~6 days (Fig. 4f and see Supplementary Fig. 16 for enlarged image). The NO concentration did not increase in proportion to the quantity of materials (block connection in Fig. 4g). On the other hand, when NaNO_2_-Mg/Al(3/1) and FeSO_4_∙7H_2_O were alternatively connected (Fig. 4g), the NO concentration could be increased in proportion to the quantity of the materials. This is because HNO_2_ generation reached saturation under chemical equilibrium (eq. 3 in Fig. 1b) in case of block connection, whereas HNO_2_ was converted to neutral NO in each step without disturbing HNO_2_ generation in the next step in case of the alternative connection.

The IR spectra of the as-prepared NaNO_2_-Mg/Al(3/1) showed an intense absorption band of NO_2_^−^ at 1227 cm^−1^ (Supplementary Fig. 16). After the LDH was exposed to exhaled breath for 2 weeks, the NO_2_^−^ signal was reduced, yet the CO_3_^2−^ signal at 1360 cm^−1^ increased. This result indicates the dominant role of the CO_2_-triggered anion-exchange reaction for HNO_2_ release. XRD analyses also supported these results (Supplementary Fig. 16).

### Battery-free respirator for inhaled NO

**Fig. 5:**
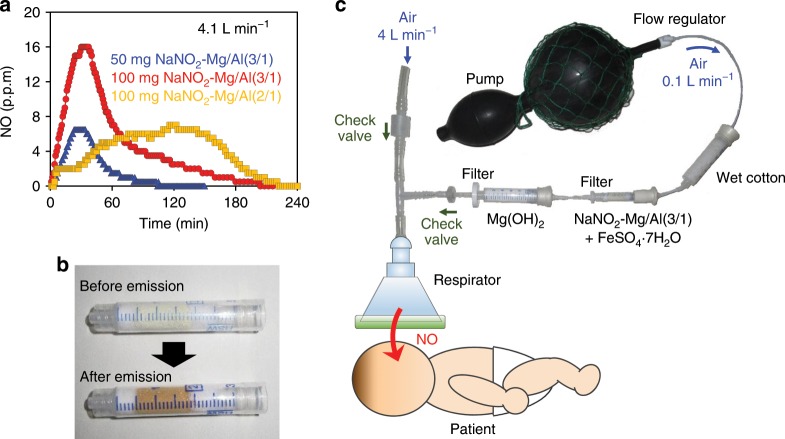
Battery-free respirator for inhaled NO. **a** Concentration of NO released from the mixture of NO_2_^−^-incorporated LDH and 10 equivalent (in weight) of FeSO_4_∙7H_2_O under 4.1 L min^−1^ air. See Supplementary Fig. 5 for experimental set-up. **b** Photograph of NaNO_2_-Mg/Al(3/1) and FeSO_4_∙7H_2_O mixture before and after ending of NO release. **c** Prototype portable and battery-free respirator for inhaled NO.

The potential utility of the gas release system was demonstrated by creating a portable and battery-free respirator that can supply therapeutically useful quantity of NO into inhaled air. NO becomes a selective and fast-acting pulmonary vasodilator upon inhalation, and inhaled NO is a well-established method for treating respiratory distress such as persistent pulmonary hypertension of the newborn^33–36^. However, current inhaled NO is an advanced medical treatment since it requires a high-pressure gas cylinder, expensive medical instrument, and trained operator for controlling/monitoring the purity and dose of NO^35,36^. The typical concentration of NO used for treatment of respiratory distress is 5–20 p.p.m.^33–36^ and the respiratory volume of newborns and infants is ~0.5–2.5 L min^−1^. Although the NO-release systems described in Fig. 4b–g, which are based on spatially isolated LDH and a reducing agent, provided NO over two weeks, limited quantity of NO (~1 p.p.m. NO, 100 mL min^−1^) could be obtained from 100 mg NaNO_2_-Mg/Al(3/1) under air. Thus, it will require gram scale of materials to satisfy the criteria (5–20 p.p.m., 0.5–2.5 L min^−1^) of inhaled NO. In contrast, the results of Fig. 4g suggest that a spontaneous conversion of HNO_2_ into NO is effective for NO accumulation (as a result of forwarding eq. 3 in Fig. 1b). Thus, we attempted to mix NaNO_2_-Mg/Al(3/1) (100 mg) and FeSO_4_∙7H_2_O (1.0 g) in powder form, and found that injection of wet air (saturated humidity, 100 mL min^−1^) to the mixture led to release of highly concentrated NO (up to 650 p.p.m.) [CAUTION!!]. It is noted that preparation of wet air (with saturated humidity) is much easier than preparing air with other fixed humidity (e.g., 10%RH). After dilution with ambient air (4.0 L min^−1^), 5–16 p.p.m. NO was obtained for about 1 h (Fig. 5a). The use of Mg(OH)_2_ effectively reduced the concentration of contaminated NO_2_ to 0.03–0.075 p.p.m., which is much lower than the permissible limit of concentration determined by the U.S. Environmental Protection Agency (=1 p.p.m.)^37^. It is noteworthy that mixing of NaNO_2_ and FeSO_4_∙7H_2_O caused a burst release of NO [CAUTION!!] due to rapid reaction of NO_2_^−^ and Fe^2+^ (Supplementary Fig. 17e).

The RH required to initiate NO release from the mixture was more than 60% (Supplementary Figs. 6 and 17a) and the NO concentration could be adjusted by manipulating the RH (Supplementary Fig. 17b). Moreover, we found that wet N_2_ is also applicable (Supplementary Fig. 17c). NO release under wet N_2_ indicates that it is not governed by CO_2_-triggered anion exchange and a plausible mechanism is that the direct anion exchange between NO_2_^−^ and SO_4_^2−^ occurred in the mixed solids in a similar way as that reported for the anion exchange of LDHs in KBr powder^38^ (Supplementary Fig. 18a). Accordingly, self-reactive Fe(NO_2_)_2_ was formed outside LDHs, and then NO_2_^−^ was reduced to NO by Fe^2+^. This hypothesis is supported by powder XRD patterns of post-release mixtures of NaNO_2_-Mg/Al(3/1) and FeSO_4_∙7H_2_O, showing typical XRD patterns of SO_4_^2−^-type LDH (Supplementary Fig. 18b)^39^.

As shown in Fig. 5a, the concentration of NO release can be controlled by adjusting the amount of NaNO_2_-Mg/Al(3/1). Moreover, the duration of NO release is elongated using NaNO_2_-Mg/Al(2/1). After the release of NO, the mixture changed its color from aqua-blue to brown, implying the oxidation of Fe^2+^ to Fe^3+^ (Fig. 5b). The NO generation was further confirmed by other methods, including gas-phase IR spectroscopy and chemiluminescence (Supplementary Figs. 7, 8, 19, and 20). Moreover, NO_2_^−^-incorporated LDHs were stable at RT as long as they were kept isolated from air (Supplementary Fig. 17d).

Finally, we constructed a completely hand-operated (i.e., battery-free), disposable, and maintenance-free apparatus that can supply NO into the respirator (Fig. 5c). Wet air flow (~100 mL min^−1^) was delivered to the mixture of NaNO_2_-Mg/Al(3/1) and FeSO_4_∙7H_2_O using a hand pump and humidifier (wet cotton). After passing through Mg(OH)_2_, purified NO was mixed into the main air stream of the respirator. The NO concentration measured by the electrochemical sensor at the respirator was consistent with the result shown in Fig. 5a. Besides the merits described before, following are the notable technical features of our gas delivery system: (i) low risk of overdose (as far as LDH amount is adequate) and (ii) visibility of gas generation (through Fe^3+^ formation). In addition, if gas is released under N_2_ flow, the obtained gas can be stored for a while without being oxidized.

In conclusion, solid materials that release p.p.m.-level H_2_S and NO in response to aerial components (CO_2_ and H_2_O) are developed based on anion-exchange properties of LDHs at the solid–gas and solid–solid interfaces. The concentration and duration of gas release are controllable by adjusting various factors (composition of materials, diffusion of gas molecules and anions, and chemical equilibrium). Not only protic gases (i.e., H_2_S and HNO_2_) but also nonprotic gases (i.e., NO and NO_2_) can be released by combining anion-exchange and redox reactions. The concept is generally applicable and will guide a design of controlled release of various types of gases using a CO_2_- and H_2_O-affinitive confined interlayer space as a reaction vessel. A CO_2_-stimulus is safe, free of cost, ubiquitous and nearly constant in concentration (~400 p.p.m.) on earth; hence, LDH-based gas-release materials is advantageous for operation under ambient air and will expand opportunities of utilizing functional gases in society, including application of physiologically active gases in developing countries and outside hospital.

### Reporting summary

Further information on experimental design is available in the Nature Research Reporting Summary linked to this paper.

## Supplementary information

Supplementary Information for Publication

Reporting summary

## Data Availability

The data that support the finding of this study are available from the corresponding authors upon reasonable request.
