# Supplementary material for: Controlled release of H2S and NO gases through CO2-stimulated anion exchange
Source: Nat Commun. 2020 Jan 23;11:453. doi: 10.1038/s41467-019-14270-3 (PMC6978355; doi:10.1038/s41467-019-14270-3)
Supplement: Supplementary file 1 — Supplementary Information for Publication [file 41467_2019_14270_MOESM1_ESM.pdf]

Supplementary Information

Controlled Release of H_2S and NO Gases Through CO_2-Stimulated Anion Exchange

Ishihara *et al.*

Contents:

Supplementary Methods

Materials....3

General methods....3

Syntheses of LDHs....4

Monitoring H_2S....6

Monitoring NO_x....6

Supplementary Table

Supplementary Table 1....8

Supplementary Figures

Supplementary Figure 1....9

Supplementary Figure 2....9

Supplementary Figure 3....10

Supplementary Figure 4....10

Supplementary Figure 5....11

Supplementary Figure 6....11

Supplementary Figure 7....12

Supplementary Figure 8....12

Supplementary Figure 9....13

Supplementary Figure 10....13

Supplementary Figure 11....14

Supplementary Figure 12....15

Supplementary Figure 13....16

Supplementary Figure 14....16

Supplementary Figure 15....17

Supplementary Figure 16....18

Supplementary Figure 17....19

Supplementary Figure 18....20

Supplementary Figure 19....20

Supplementary Figure 20....21

Supplementary Figure 21....22

Supplementary Figure 22....23

Supplementary Figure 23....23

Supplementary Discussion

Quantification of HS^- involved in LDH....24

Quantification of NO_2^- involved in LDH....25

Additional discussion on H_2S release profile....25

Supplementary Methods

Materials.

$NaHS\cdot nH_2O$ (65%), $Na_2S\cdot 9H_2O$, and $NaNO_2$ were obtained from Wako Pure Chemical Industries, Ltd. Granular $FeSO_4\cdot 7H_2O$ (Fujifilm Wako Pure Chemical Corp.) was ground on a mortar to prepare powdered samples. $Mg(OH)_2$, 0.1 mol L^{-1} HCl in ethanol, and 3% HCl in ethanol were purchased from Kanto Chemical Co., Inc. Methanol, PbS, and $Pb(CH_3COO)_2\cdot 3H_2O$ were obtained from Nakalai Tesque, Inc. Griess reagent (Sigma-Aldrich) was used as received. The gas cylinder (compressed dry air, CO_2, 25.1 ppm H_2S in N_2, 24.9 ppm NO in N_2) was obtained from Suzuki Shokan Co., Ltd. Water was deionized using PURELAB Option-S7/15 (ELGA), and then degassed by boiling for 15 min under N_2 bubbling (0.1–0.3 L min^{-1}), followed by cooling down to RT under the same N_2-bubbling condition. Degassed deionized water was stored in a sealed glass bottle and used for synthesis of LDHs. $Mg_3Al(OH)_8(CO_3^{2-})_{0.5}\cdot 2H_2O$ was purchased from Kyowa Kagaku Kogyo Co., Ltd. $Mg_2Al(OH)_6(CO_3^{2-})_{0.5}\cdot 2H_2O$ was prepared by a hydrothermal reaction according to literature procedure.[25] $Mg_3Al(OH)_8(Cl^-)\cdot 2H_2O$ and $Mg_2Al(OH)_6(Cl^-)\cdot 2H_2O$ were prepared by an anion-exchange reaction according to a previously reported procedure.[16,26] A porous tape (KEEP PORE™, 25 mm × 8 m) was obtained from Nichiban Co., Ltd. A hydrophilic PTFE membrane filter (Omnipore™ membrane filter JGWP04700, pore size = 0.2 µm, diameter = 47 mm) obtained from Merck Millipore, Ltd., was utilized for filtration of LDH products. A small syringe filter (Cosmonice filter S, Pore size = 0.45 µm, Filter diameter = 13 mm) was purchased from Nakalai Tesque, Inc. A large syringe filter (PFSF-2545PT, pore size = 0.45 µm, filter diameter = 25 mm) was purchased from AS ONE Corporation.

General methods.

FT-IR spectra of the powdered sample were measured by IR Affinity-1 (Shimadzu) in ATR mode, as well as by Spectrum One FT-IR apparatus with ATR attachment (Perkin-Elmer). Powder XRD was measured by RINT2200V (Rigaku Co., Ltd.) with CuK_α at a scan rate of 2 ° min^{-1} under dry N_2-flow. TG-DTA was measured by ThermoPlus TG8120 (Rigaku Co., Ltd.) at a heating rate of 10 °C min^{-1} under air flow (20 mL min^{-1}). Approximately 10 mg of LDH in a Pt pan was measured, and α-Al_2O_3 was used as a standard. The SEM image was monitored by S-4800 (Hitachi) at 10 kV. The energy-dispersive X-ray spectrometry (EDS) spectrum was measured by SEM-EDS apparatus (JEOL, JSM6010LA) at an acceleration voltage of 10−15 kV. Powdery LDH on the carbon tape was monitored without coating of conductive layers. The electric absorption spectrum was measured using UV-3600 (Shimadzu) at RT. CO_2 was monitored by TESTO 535 (TESTO) within a detection range of 0−9999 ppm (resolution = 1 ppm). O_2 was monitored by a digital sensor (Oxy-M and Oxy-1S-M, ICHINEN JIKCO., Ltd.). RH was monitored by HMI-41 (VAISALA). The flow rate was monitored by a float-ball-type flow meter (KOFLOCK) or a digital flow meter (7000 Flowmeter, Ellutia). A digital camera in the time-lapse mode (EX-ZR1800, CASHIO) was utilized for recording values of gas sensors every 1−5 min. All detector tubes (No.4L for H_2S, No.5Lb for SO_2, No.9P for NO_2, No.11L for NO+NO_2, and No.10 for separate quantification of NO and NO_2) were purchased from GASTEC. Sampling of gases using the detector tube was performed with a handy pump (GV-100S, GASTEC) or a battery-powered

pump (GSP-300FT-2 or GSP-400FT, GASTEC). An electric oven (IW-300S or AVO-250NB, ETTAS) was used to heat samples at constant temperature.

Syntheses of LDHs.

Syntheses of NaHS-Mg/Al(2/1) and NaHS-Mg/Al(3/1)

All experiments (except for sonication) were performed under dry N_2 using a globe box. To 40 mg of $Mg_2Al(OH)_6(Cl^-)\cdot 2H_2O$ or 50 mg of $Mg_3Al(OH)_8(Cl^-)\cdot 2H_2O$ in a screw-cap glass vial (50 mL), degassed deionized water (22.8 mL) was added, and then, 7.2 mL of $NaHS\cdot nH_2O$ dissolved in degassed deionized water (19.5 mg mL^{-1}). After closing the screw cap tightly, the glass bottle (taken out from globe box) was soaked in an ultrasonication bath for dispersion of LDH particles (for ~30 s). The glass bottle was stored at RT under N_2 for 2 days. The suspension was filtered on the PTFE membrane filter (Omnipore™, pore size = 0.2 μm) and washed with degassed deionized water (2 mL × 5 times). The membrane filter with the sample was cut to a desired size (e.g., semicircle and quadrant), and dried in vacuum for 3 h. The solid sample on the membrane filter was kept in a screw-cap glass vial (13.5 mL), which, if necessary, was stored in a gas barrier bag (Lamizip® AL-D, Seisannipponsha, Ltd.). Yield: quantitative. When changing the amount of $NaHS\cdot nH_2O$, the mixing ratio of degassed deionized water and aqueous solution of $NaHS\cdot nH_2O$ (19.5 mg mL^{-1}) was varied for maintaining the total volume of the solution at 30 mL.

Syntheses of Na_2S-Mg/Al(2/1) and Na_2S-Mg/Al(3/1)

All experiments (except for sonication) were performed under dry N_2 using a globe box. To 40 mg of $Mg_2Al(OH)_6(Cl^-)\cdot 2H_2O$ or 50 mg of $Mg_3Al(OH)_8(Cl^-)\cdot 2H_2O$ in the screw-cap glass vial (50 mL), degassed deionized water (27.2 mL) was added, and then, 2.8 mL of $Na_2S\cdot 9H_2O$ dissolved in degassed deionized water (139.3 mg mL^{-1}). After closing the screw cap tightly, the glass bottle (taken out from globe box) was soaked in an ultrasonication bath for dispersion of LDH particles (for ~30 s). The glass bottle was stored at room temperature under N_2 for 2 days. The suspension was filtered on the PTFE membrane filter (Omnipore™, pore size = 0.2 μm) and washed with degassed deionized water (2 mL × 5 times). The membrane filter with the sample was cut to the desired size (e.g., semicircle and quadrant), and dried in vacuum for 3 h. The solid sample on the membrane filter was kept in the screw-cap glass vial (13.5 mL), which if necessary, was stored in gas barrier bag (Lamizip® AL-D, Seisannipponsha, Ltd.). Yield: quantitative.

Syntheses of NaHS-Mg/Al(2/1) in methanol

All experiments (except for sonication) were performed under dry N_2 using a globe box. To 20 mg of $Mg_2Al(OH)_6(Cl^-)\cdot 2H_2O$ in the screw-cap glass vial (40 mL), degassed methanol (20 mL) containing 7.25 mg of $NaHS\cdot nH_2O$ was added. Two batches were prepared. After closing the screw cap tightly, the glass bottle (taken out from the globe box) was soaked in an ultrasonication bath for dispersion of LDH particles until fully dispersed suspensions were formed. The two glass bottles were stored at RT under N_2 for 2 days. The suspension in one vessel was filtered on the PTFE membrane filter (Omnipore™, pore size = 0.2 μm) and washed with degassed methanol. Another suspension was filtered on the PTFE membrane filter (pore size = 0.2 μm) and washed with degassed deionized water of the same amount with MeOH. Each membrane filter was folded to a semicircle (with sample inside), and then cut into four fan-shaped isometric pieces. Each piece

containing 5.0 mg NaHS-Mg/Al(2/1) was put into a glass vial, and dried in vacuum for 40 min. Each sample was kept in a screw-cap glass vial (13.5 mL). These samples were utilized only for discussing Supplementary Fig. 23.

Small-scale syntheses of $NaNO_2$-Mg/Al(2/1) and $NaNO_2$-Mg/Al(3/1) in globe box

All experiments (except for sonication) were performed under dry N_2 using a globe box. To 40 mg of $Mg_2Al(OH)_6(Cl^-)\cdot 2H_2O$ or $Mg_3Al(OH)_8(Cl^-)\cdot 2H_2O$ in a screw-cap glass vial (50 mL), degassed deionized water (28.2 mL) was added, and then, 1.8 mL of $NaNO_2$ dissolved in degassed deionized water (100 mg mL^{-1}). After closing the screw cap tightly, the glass bottle (taken out from the globe box) was soaked in an ultrasonication bath for dispersion of LDH particles (for ~30 s). The glass bottle was returned to the globe box and stored at RT under N_2 for 2 days. The suspension was filtered on the PTFE membrane filter (Omnipore™, pore size = 0.2 μm), and washed with degassed deionized water (2 mL × 5 times). The membrane filter with the sample was cut to the desired size (e.g., semicircle and quadrant), and dried in vacuum for 3 h. The white solid sample on the membrane filter was kept in a screw-cap glass vial (13.5 mL), which if necessary, was stored in a gas barrier bag (Lamizip® AL-D, Seisannipponsha, Ltd.). Yield: quantitative. When changing the amount of $NaNO_2$, the mixing ratio of degassed deionized water and aqueous solution of $NaNO_2$ (100 mg mL^{-1}) was varied for maintaining the total volume of the solution as 30 mL. The amount of $NaNO_2$ used in the synthesis of $NaNO_2$-Mg/Al(3/1) was optimized by comparing three conditions (36.5, 183, or 914 mg of $NaNO_2$ for 40 mg $Mg_3Al(OH)_8(Cl^-)\cdot 2H_2O$). $NaNO_2$-Mg/Al(3/1) prepared from 183 mg $NaNO_2$ demonstrated 0.5 ppm of NO + NO_2 + HNO_2 (tested by detector tube, GASTEC−11L) after exposure to air for 30 min. In contrast, $NaNO_2$-Mg/Al(3/1) prepared from 36.5 mg $NaNO_2$ and 914 mg $NaNO_2$ demonstrated 0.4 ppm and 0.25 ppm of NO + NO_2 + HNO_2, respectively. Thus, 183 mg $NaNO_2$ (20.4 equivalent in mole for Cl^-) was found as the optimal mixing amount for 40 mg of $Mg_3Al(OH)_8(Cl^-)\cdot 2H_2O$. As NO release experiments require gram scale of NO_2^--incorporated LDHs in total, we chose $NaNO_2$-Mg/Al(3/1) for the main study. $Mg_3Al(OH)_8(CO_3^{2-})_{0.5}\cdot 2H_2O$ is commercially available on the kilogram scale, but $Mg_2Al(OH)_6(CO_3^{2-})_{0.5}\cdot 2H_2O$ must be synthesized by a hydrothermal reaction.

Large-scale synthesis of $NaNO_2$-Mg/Al(3/1) without globe box

To 2.0 g of $Mg_3Al(OH)_8(Cl^-)\cdot 2H_2O$ in a round-bottom three-neck flask (500 mL), degassed deionized water (300 mL) was added after purging the flask with dry N_2. The flask was soaked in an ultrasonication bath for dispersion of LDH particles (for ~3 min). Then, 9.5 g of $NaNO_2$ dissolved in degassed deionized water (40 mL) was added to the solution using a needle syringe through a rubber septum. The suspension was stirred under N_2 for one day, and then, left standing without stirring for another day. The suspension was filtered on a PTFE membrane filter (Omnipore™, pore size = 0.2 μm) under N_2, washed with degassed deionized water (10 mL × 3 times) and methanol (10 mL × 1), and dried in vacuum at 40 °C for 5 h. The white solid sample on the membrane filter was kept in a screw-cap glass vial (13.5 mL). The sample was stable over months when kept in the screw-cap glass vial. Yield: 1.9 g. $NaNO_2$-Mg/Al(3/1) prepared in this method shows an identical IR spectrum to that of $NaNO_2$-Mg/Al(3/1) prepared in the globe box.

Monitoring H_2S.

Precipitation of Pb^{2+}

We bubbled 38 mg of $Pb(CH_3COO)_2 \cdot 3H_2O$ dissolved in 10 mL distilled H_2O with H_2S released from four patches of NaHS-Mg/Al(2/1) (Fig. 3a) under ambient air flow (100 mL min^{-1}, 76%RH). After 1 h, the formed black precipitate was collected by centrifugation, followed by washing with water, diluted acetic acid (0.05 mL acetic acid in 20 mL H_2O), and methanol. The use of dilute acetic acid was effective in removing the white precipitate ($Pd(CO_3)$ and/or $Pd(OH)_2$) that appeared even when $Pb(CH_3COO)_2$ aqueous solution was bubbled with air. The black precipitate was dispersed in methanol, and the suspension was spread on a glass plate. After drying methanol, powder XRD was measured. PbS standard was also dispersed in methanol, and the suspension was spread on a glass plate. After drying methanol, powder XRD was measured.

Detector tube

Detector tubes for H_2S (GASTEC–4L) and SO_2 (GASTEC–5Lb) were utilized for quantitative monitoring of the corresponding gases.

Gas sensor

H_2S was monitored using an electrochemical sensor (ToxiRAE 3, RAE SYSTEMS) within a detection range of 0.4–100 ppm (resolution = 0.1 ppm). The H_2S electrochemical sensor was calibrated by standard gas (25.1 ppm H_2S in N_2). Compressed dry air (containing CO_2 and other minor components of atmospheric air) was supplied from the gas cylinder, and humidity was adjusted as shown in Supplementary Fig. 1. Dry N_2 containing adjusted amount of CO_2 was delivered as shown in Supplementary Fig. 2 using a Tedlar® bag and an electric pump (GSP-400FT, GASTEC). Unless noted, all gas release experiments were performed after aging the LDH samples attached on the membrane filter for about 1 week at RT (to reduce aging effect in sample-to-sample comparison). The volume of the glass vial used for LDH storage was 13.5 mL.

Monitoring NO_x.

Griess reagent

An aqueous solution of Griess reagent (NO^- indicator) was prepared by dissolving 1.0 g Griess reagent (Sigma-Aldrich) in 25 mL of distilled water. Then, 100 mL min^{-1} of ambient air (20 °C, 35%RH) was passed through the glass vial containing 100 mg $NaNO_2$-Mg/Al(3/1), followed by bubbling of the gas into the aqueous solution of the Griess reagent (3 mL). After 15 min bubbling, the absorption spectra of the solution were measured using a 1 cm quarts cell. Note that the Griess reagent also responds to NO_2 as NO_2 produces HNO_2 in water ($2NO_2 + H_2O \rightarrow HNO_3 + HNO_2$).

Detector tube

NO, NO_2, and HNO_2 were monitored by combining several detector tubes. Humidity-controlled air or exhaled breath (involving 4.0% CO_2 and saturated humidity) was supplied by an electric pump to 100 mg $NaNO_2$-Mg/Al(3/1), as shown in Supplementary Fig. 3. When necessary, granular $FeSO_4 \cdot 7H_2O$ (~750 mg, 3 cm in length) and powdery $Mg(OH)_2$ (~250 mg, 3 cm in length) loaded in the glass tube were inserted into the flow line for converting HNO_2 to NO and removing NO_2, respectively. As the permissible humidity range of the detector tubes was 0–90%, the sampling gas was diluted with the same amount of dry air (i.e., RH was

adjusted to ~50%RH upon monitoring with the detector tube). Therefore, the actual concentration of analyte gases was twice the value shown by the detector tubes. The NO_2 concentration was determined using GASTEC−9P, which is insensitive to HNO_2 and NO. The gas sampling rate was 100 mL min^{-1}. Concentration of NO + NO_2 + HNO_2 was determined using GASTEC−11L, which involved a strong oxidant (Cr^{3+} + H_2SO_4) at the entry for converting NO to NO_2. HNO_2 should be oxidized to NO_2 as well. Then, the resulting NO_2 was quantified by *o*-tolidine (aromatic amine), a NO_2 indicator. The standard sampling rate for GASTEC−11L was 50 mL min^{-1}, so that excess gas was vented outside using the check valve. The NO concentration was determined by combining two detector tubes, GASTEC−10 (for NO_2) and GASTEC−11L, as shown in Supplementary Fig. 3. NO_2 and HNO_2 were removed by the first detector tube for NO_2 (GASTEC−10), which contains *o*-tolidine. NO could pass through this tube, and then was detected by the second detector tube (GASTEC−11L) after conversion to NO_2. The HNO_2 concentration was estimated by subtracting NO and NO_2 from NO + NO_2 + HNO_2.

Gas sensor

NO was monitored by a digital sensor (ToxiRAE Pro, RAE SYSTEMS) within a detection range of 0.5−250 ppm (resolution = 0.5 ppm). The NO digital sensor was calibrated by standard gas (24.9 ppm NO in N_2). Exhaled breath (containing 4.0% CO_2 and saturated relative humidity) in a Tedlar® bag was delivered by an electrical pump, as shown in Supplementary Fig. 4. HNO_2 was converted to NO using granular $FeSO_4{\cdot}7H_2O$ (~750 mg, 3 cm in length), and monitored by a NO sensor. $FeSO_4{\cdot}7H_2O$ was occasionally changed to new ones.

Release of NO from a mixture of 100 mg $NaNO_2$-Mg/Al(3/1) and 1.0 g $FeSO_4{\cdot}7H_2O$ was monitored as shown in Supplementary Fig. 5. Humid air (100 mL min^{-1}) was delivered to the mixture, and then, contaminated NO_2 was removed by 4.0 g of $Mg(OH)_2$ loaded in a 12 mL plastic syringe. NO was diluted with 4.0 L min^{-1} air and monitored by an electrochemical sensor (ToxiRAE Pro, RAE SYSTEMS). The NO_2 concentration was occasionally monitored by a detector tube (GASTEC−9P).

IR spectroscopy

NO demonstrates a characteristic IR spectrum at around 1800−1900 cm^{-1}. To exclude strong IR signals from CO_2 and H_2O, NO detection was performed under dry N_2, as shown in Supplementary Fig. 7. N_2 humidified with wet cotton was delivered to the mixture of $NaNO_2$-Mg/Al(3/1) and $FeSO_4{\cdot}7H_2O$. NO_2 was removed with $Mg(OH)_2$, and then, water was removed with molecular sieve 3A for protecting the NaCl window. The dried N_2 involving NO was injected into a gas cell (GL Science, 10 cm optical length, NaCl windows), and the IR spectrum was monitored using an FT-IR spectrometer (Nicolet, NEXUS 670-FT-IR).

Chemiluminescence

NO demonstrates characteristic chemiluminescent reaction with O_3 ($NO + O_3 \rightarrow NO_2 + O_2 + h\nu$), which is absolutely selective to NO over other NO_x. To avoid aerial oxidation of NO during the analysis, NO detection was performed under N_2, as shown in Supplementary Fig. 8. N_2 humidified with wet cotton was delivered to $NaNO_2$-Mg/Al(3/1) and $FeSO_4{\cdot}7H_2O$ mixture. NO_2 was removed with $Mg(OH)_2$, and then, the analyte gas was injected into chemiluminescent NO/NO_x analyzer (Shimadzu, NOA-7000) equipped with a pretreatment chiller (for removal of water) and solenoid valve (for switching NO/NO_x detection modes).

Supplementary Table

Supplementary Table 1. Syntheses of LDH-based H_2S-releasing materials.

Product name	LDH precursor (mg, mmol)	Sulfide reagent (mg, mmol)	Peak of H_2S release[#]
NaHS-Mg/Al(2/1)	$Mg_2Al(OH)_6(Cl^-)\cdot 2H_2O$ (40 mg, 0.16 mmol)	NaHS·$n$$H_2O$ (141 mg, 1.63 mmol)	12.6 ppm at 52 min
NaHS-Mg/Al(3/1)	$Mg_3Al(OH)_8(Cl^-)\cdot 2H_2O$ (50 mg, 0.16 mmol)	NaHS·$n$$H_2O$ (141 mg, 1.63 mmol)	25.4 ppm at 1 min
Na_2S-Mg/Al(2/1)	$Mg_2Al(OH)_6(Cl^-)\cdot 2H_2O$ (40 mg, 0.16mmol)	$Na_2S\cdot 9H_2O$ (393 mg, 1.63 mmol)	No emission
Na_2S-Mg/Al(3/1)	$Mg_3Al(OH)_8(Cl^-)\cdot 2H_2O$ (50 mg, 0.16 mmol)	$Na_2S\cdot 9H_2O$ (393 mg, 1.63 mmol)	57.8 ppm at 13 min

#Data from Fig. 2e.

Supplementary Figures

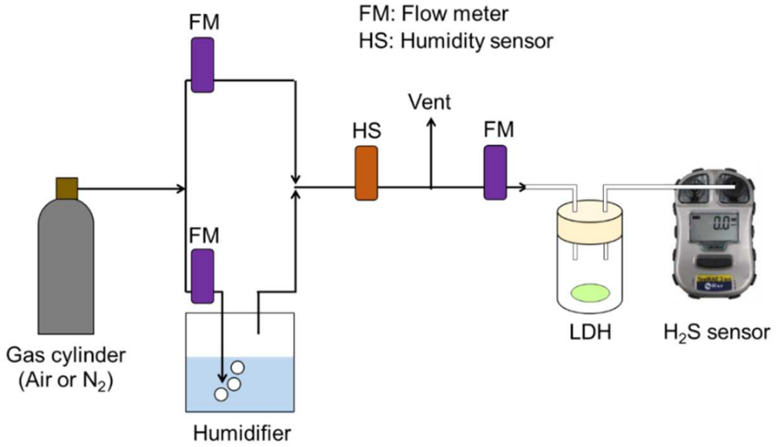

Supplementary Fig. 1. Experimental set-up. Experimental set-up for delivering carrier gas with adjusted humidity and flow rate. When necessary, LDH in glass vial was warmed in an electric oven.

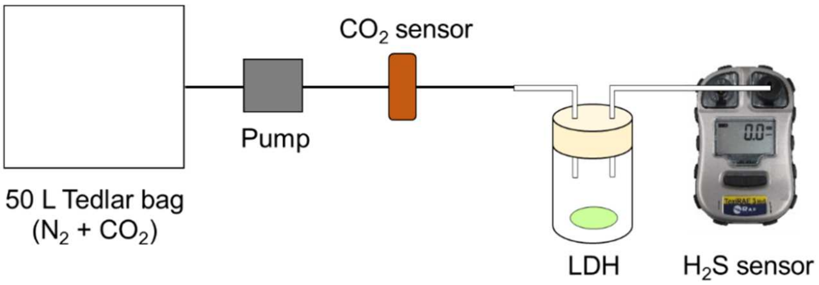

Supplementary Fig. 2. Experimental set-up. Typical experimental set-up for delivering dry N_2 containing CO_2. This experiment was performed in a globe box (dry N_2 atmosphere) for avoiding contamination of trace humidity from air. For example, 100 ppm CO_2 in N_2 was prepared by adding 5 mL CO_2 into 50 L N_2 filled in the Tedlar® bag. The volume of the glass vial used for LDH storage was 13.5 mL.

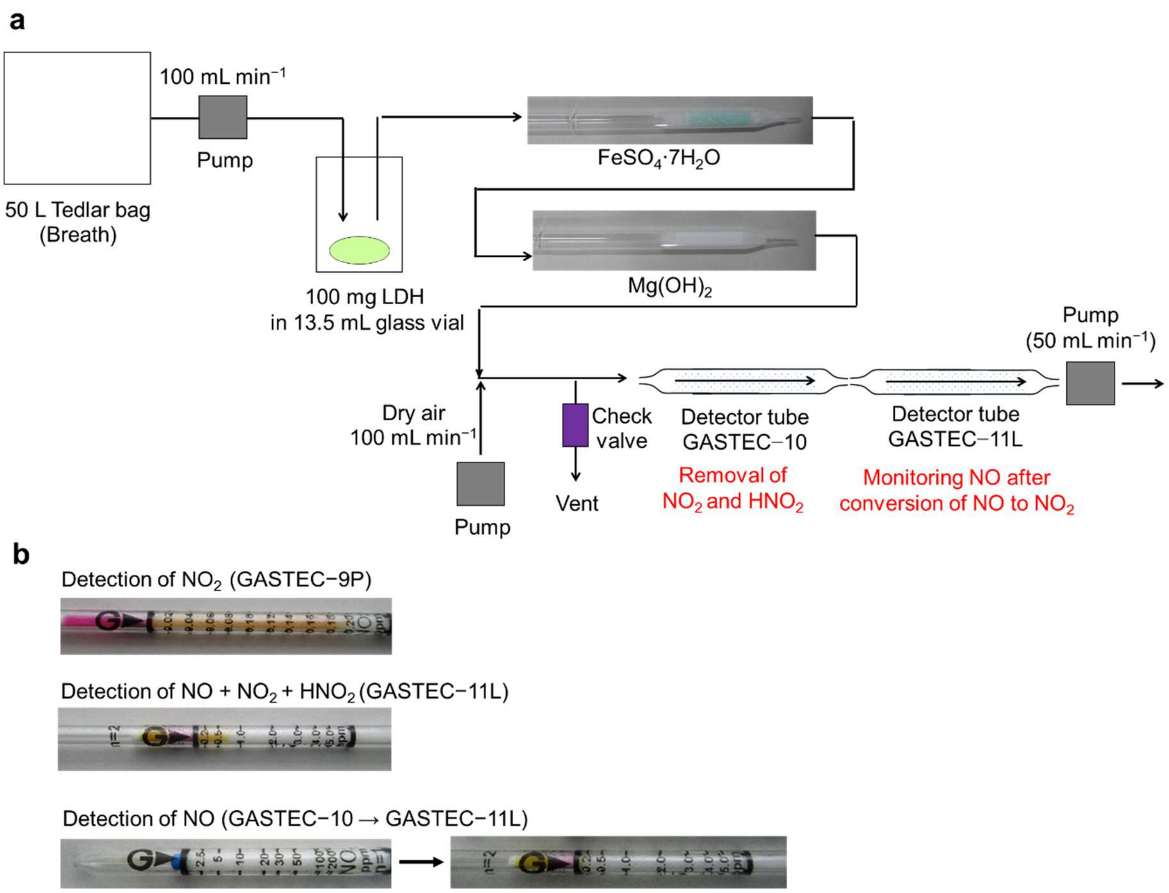

Supplementary Fig. 3. Experimental set-up. (**a**) Typical experimental set-up for monitoring NO, NO_2, and HNO_2 by detector tube. The image specifically shows NO detection. (**b**) Typical images of detector tubes responding to nitrogenous gases.

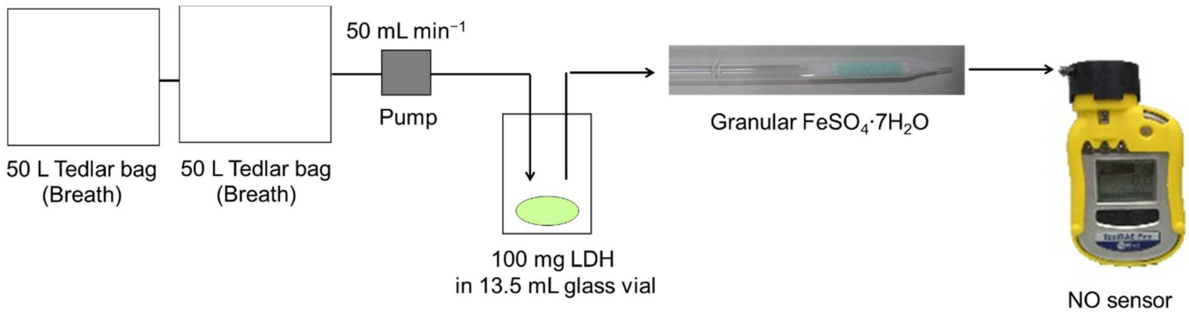

Supplementary Fig. 4. Experimental set-up. Typical experimental set-up for monitoring NO by electrochemical sensor.

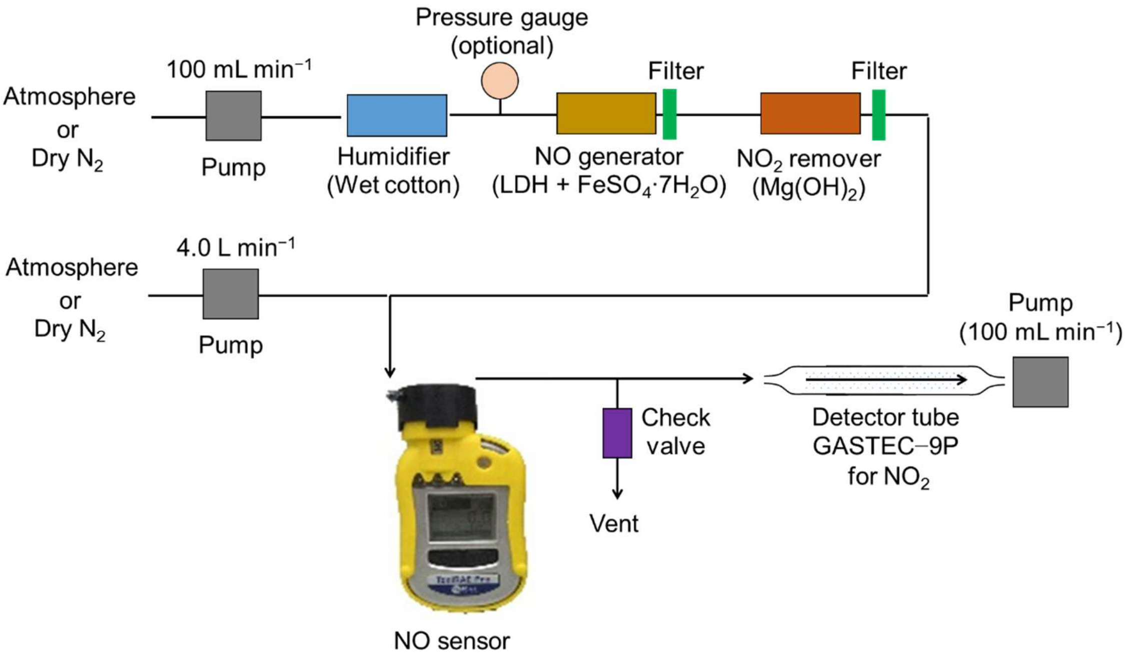

Supplementary Fig. 5. Experimental set-up. Typical experimental set-up for monitoring NO and NO_2 released from $NaNO_2$-Mg/Al(3/1) and $FeSO_4 \cdot 7H_2O$ mixture. There is a little flow resistance in the NO generator and NO_2 remover due to the presence of powdery materials, and the barometric pressure before the NO generator is measured as 0.115 MPa (i.e., 0.014 MPa higher than ambient pressure (0.101 MPa)). The use of a larger amount of $Mg(OH)_2$ can be useful for further removal of NO_2, but this will increase the flow resistance.

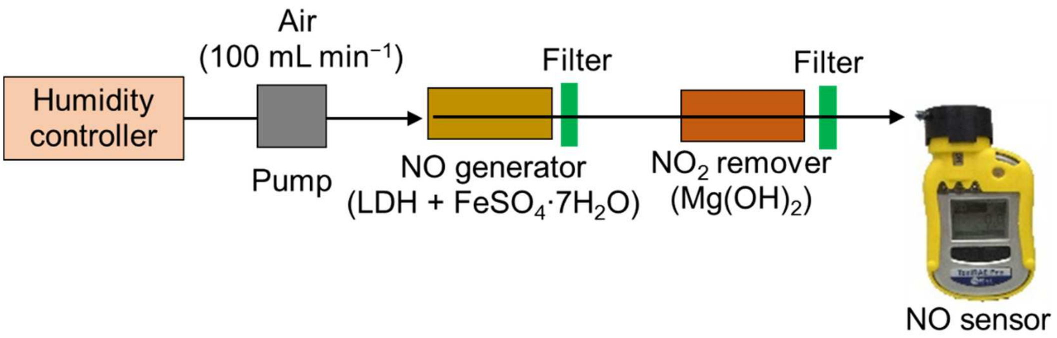

Supplementary Fig. 6. Experimental set-up. Typical experimental set-up for monitoring NO released from $NaNO_2$-Mg/Al(3/1) and $FeSO_4 \cdot 7H_2O$ mixture. As a NO_2 remover, 4.0 g of $Mg(OH)_2$ loaded in a 12 mL plastic syringe was used.

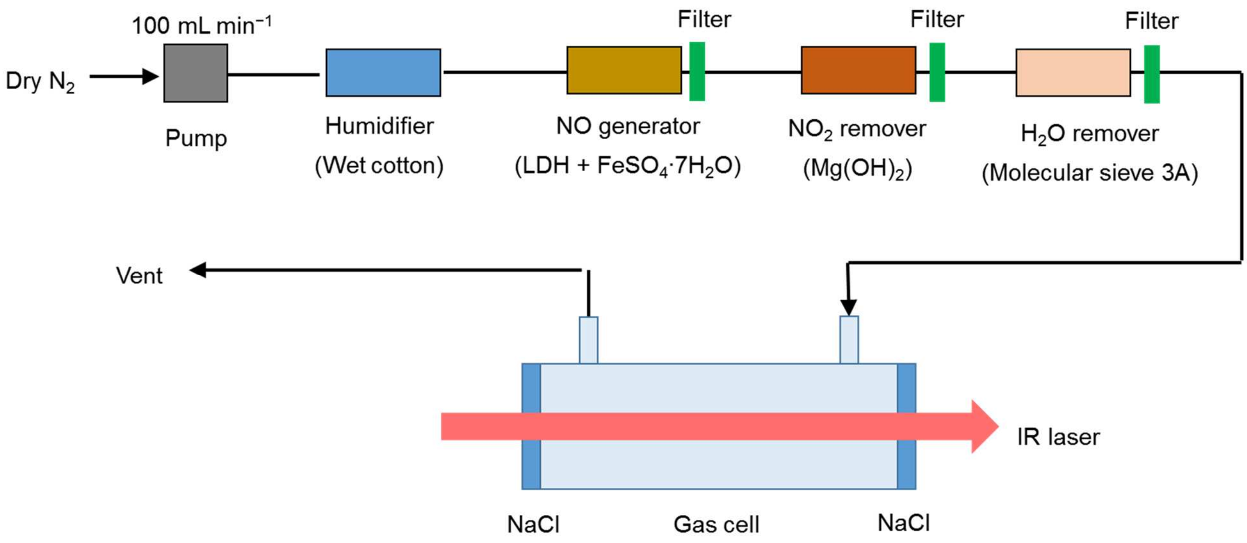

Supplementary Fig. 7. Experimental set-up. Typical experimental set-up for monitoring NO by IR spectroscopy.

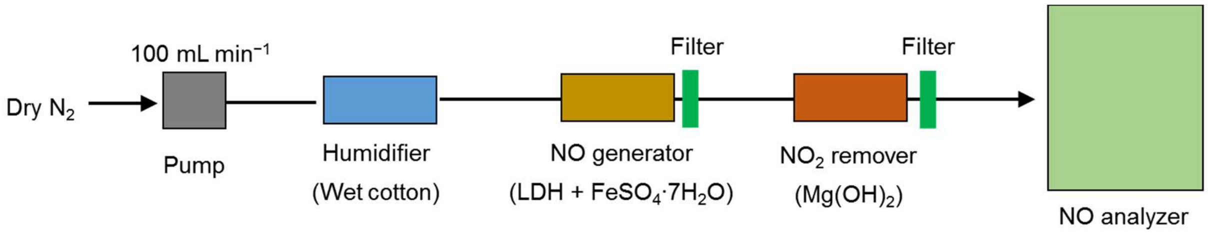

Supplementary Fig. 8. Experimental set-up. Typical experimental set-up for monitoring NO by chemiluminescence NO analyzer.

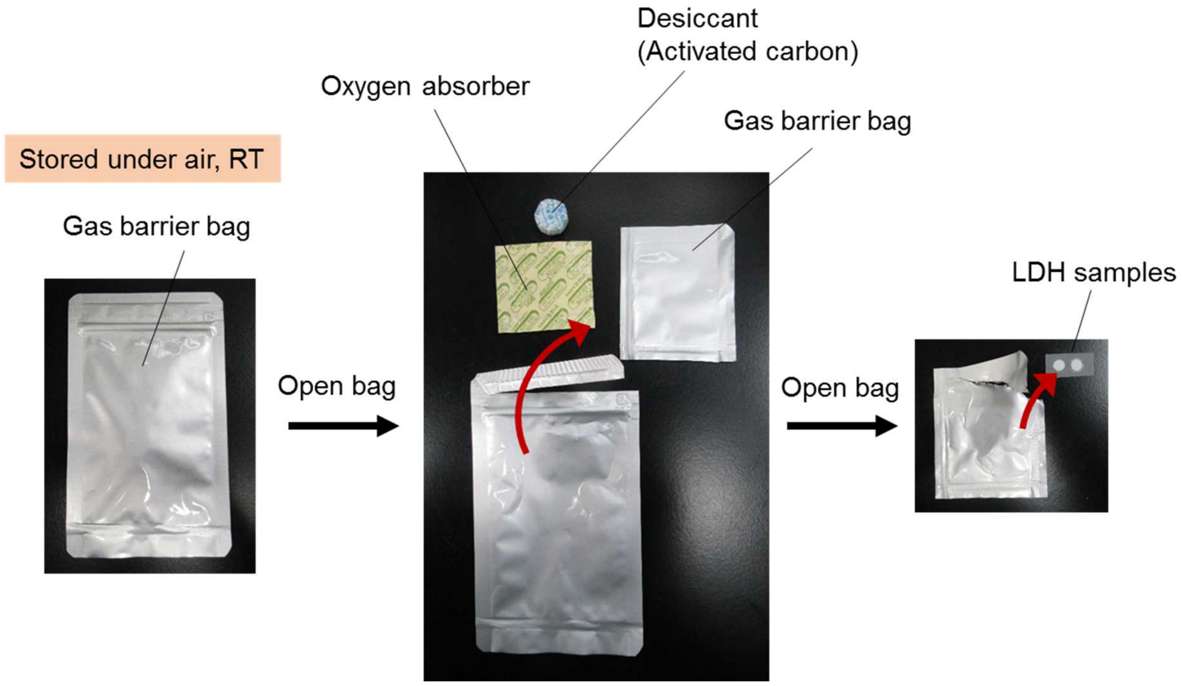

Supplementary Fig. 9. Storage of gas-releasing LDH samples. Gas barrier bag (Lamizip® AL-D, Seisannipponsha, Ltd.), desiccant + activated carbon (DO1506, As One), and oxygen absorber (AGELESS®, Mitsubishi Gas Chemical) were used to isolate LDH samples from air.

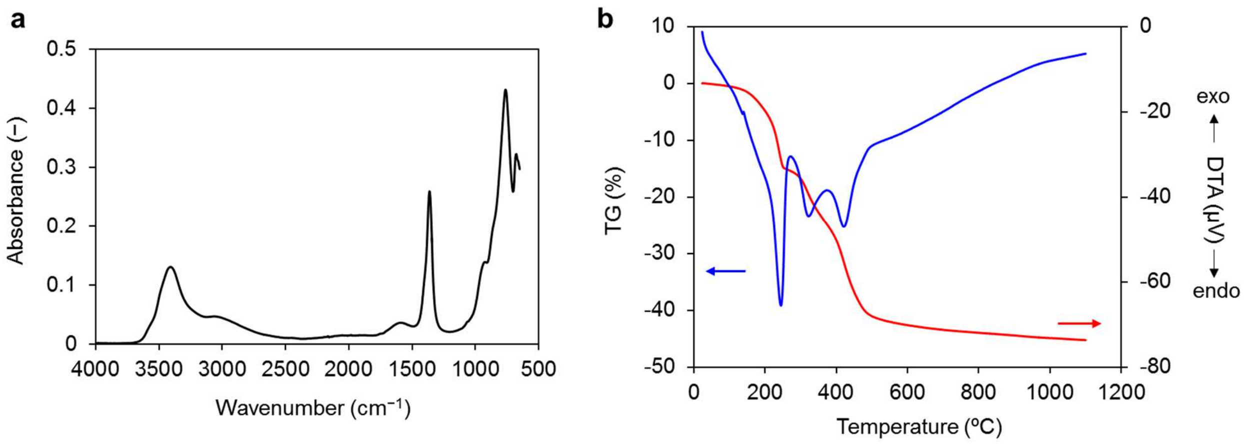

Supplementary Fig. 10. Properties of Na_2S-Mg/Al(2/1). IR spectrum (**a**) and TG-DTA profile (**b**) of as-prepared Na_2S-Mg/Al(2/1).

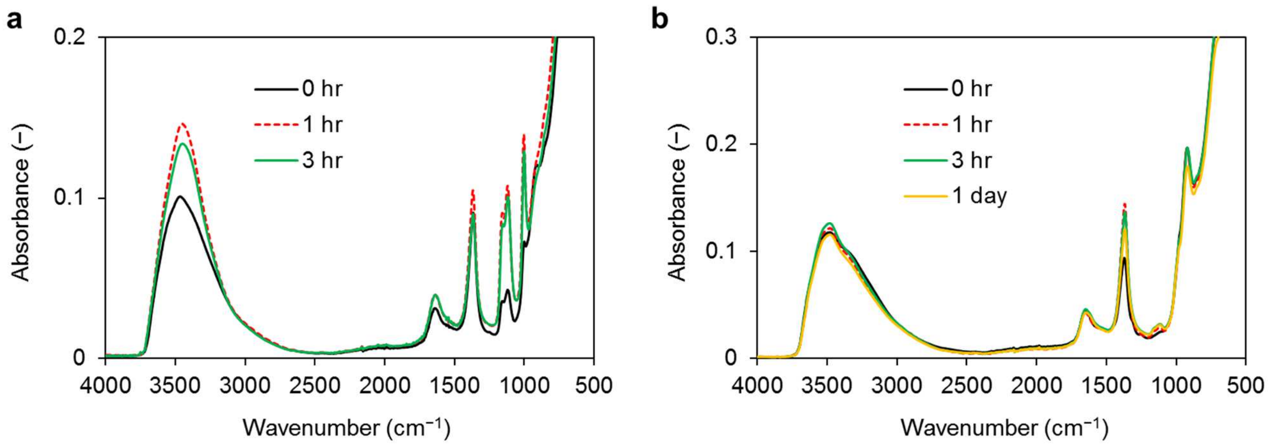

Supplementary Fig. 11. Properties of NaHS-Mg/Al(3/1) and Na_2S-Mg/Al(3/1). IR spectra of (**a**) NaHS-Mg/Al(3/1) and (**b**) Na_2S-Mg/Al(3/1) after exposure to air.

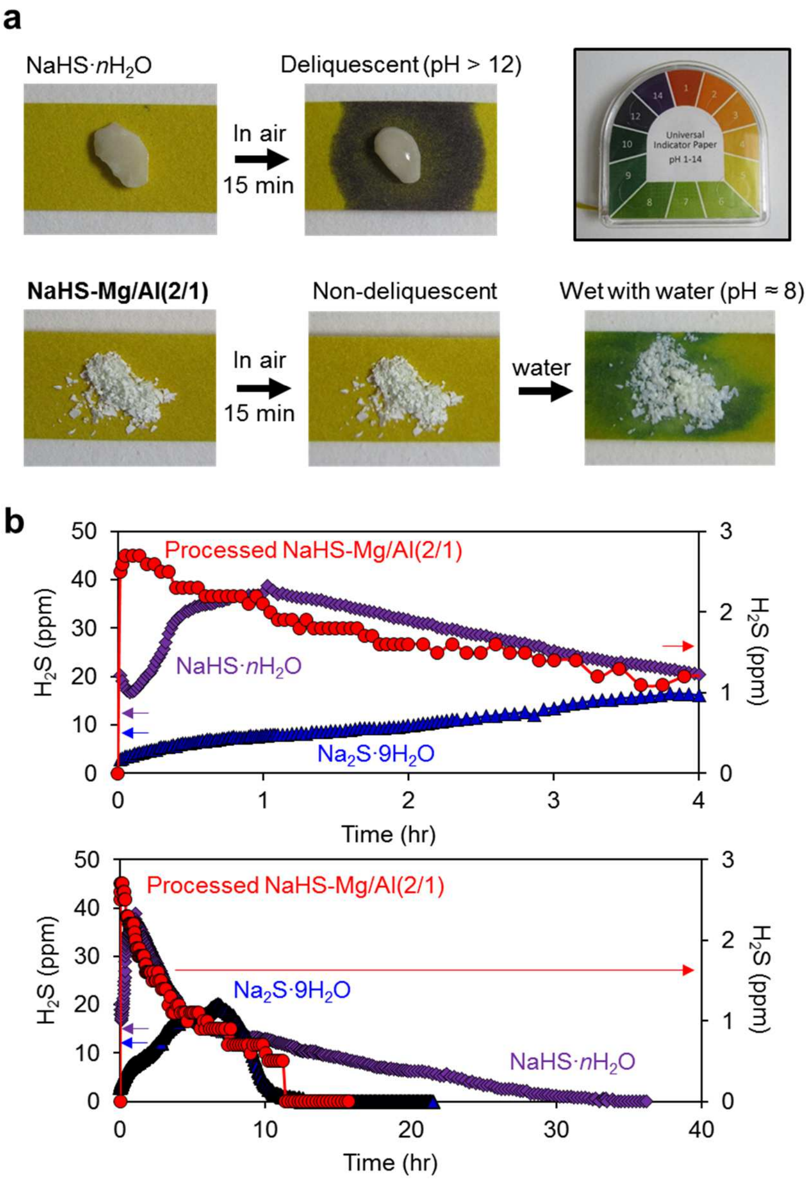

Supplementary Fig. 12. Comparison with simple salts. (a) Comparison of NaHS-Mg/Al(2/1) and NaHS in terms of handling safety (note that aqueous solutions of NaHS and Na_2S are highly basic). (b) H_2S release from powdery NaHS·*n*H_2O (8.5 mg) and $Na_2S·9H_2O$ (10.5 mg) under standard flow condition. Note that highly deliquescent NaHS·*n*H_2O changed into aqueous solution within 10 min under air, while $Na_2S·9H_2O$ maintained solid state in the condition (i.e., 50%RH). $Na_2S·9H_2O$ also exhibited deliquescence when RH was 80%. Since the aqueous solution of NaHS has small contact area with air, steady release of H_2S is reasonable. For comparison H_2S release from processed NaHS-Mg/Al(2/1) (two patches of NaHS-Mg/Al(2/1), 1.1 mg × 2) prepared by porous tape covering and heat treatment at 60 ºC for 7days (data obtained from Supplementary Fig. 15) is also shown. H_2S release from NaHS·*n*H_2O and $Na_2S·9H_2O$ was negligible under dry N_2, implying air-stimulated reaction (e.g., $2NaHS + CO_2 + H_2O \rightarrow 2H_2S + Na_2CO_3$).

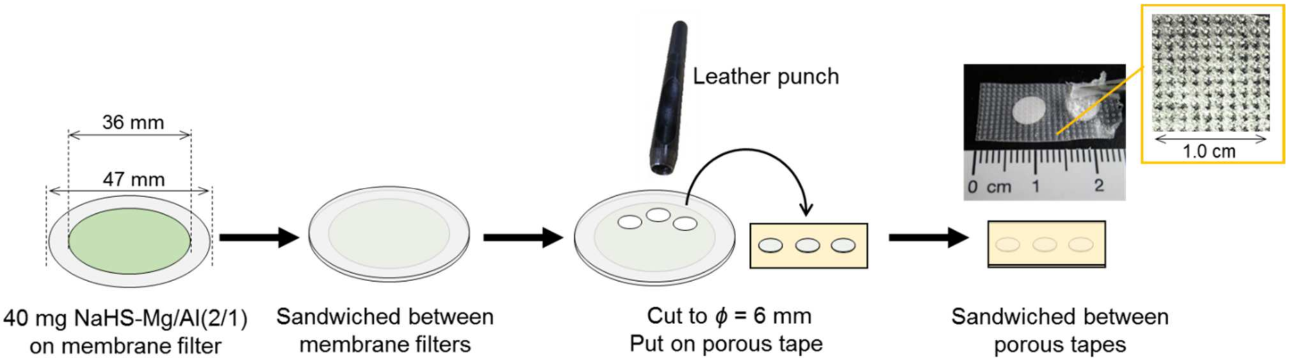

Supplementary Fig. 13. Preparation of H_2S-releasing patch. Preparation of NaHS-Mg/Al(2/1) sandwiched between porous tapes (pore size = ~0.3 mm), yielding ~20 pieces of patches containing 1.1 mg NaHS-Mg/Al(2/1). Microscopy image of the porous tape is shown in the inset. Membrane filter (pore size = 0.2 μm) is identical to that utilized for filtration of LDHs in the syntheses.

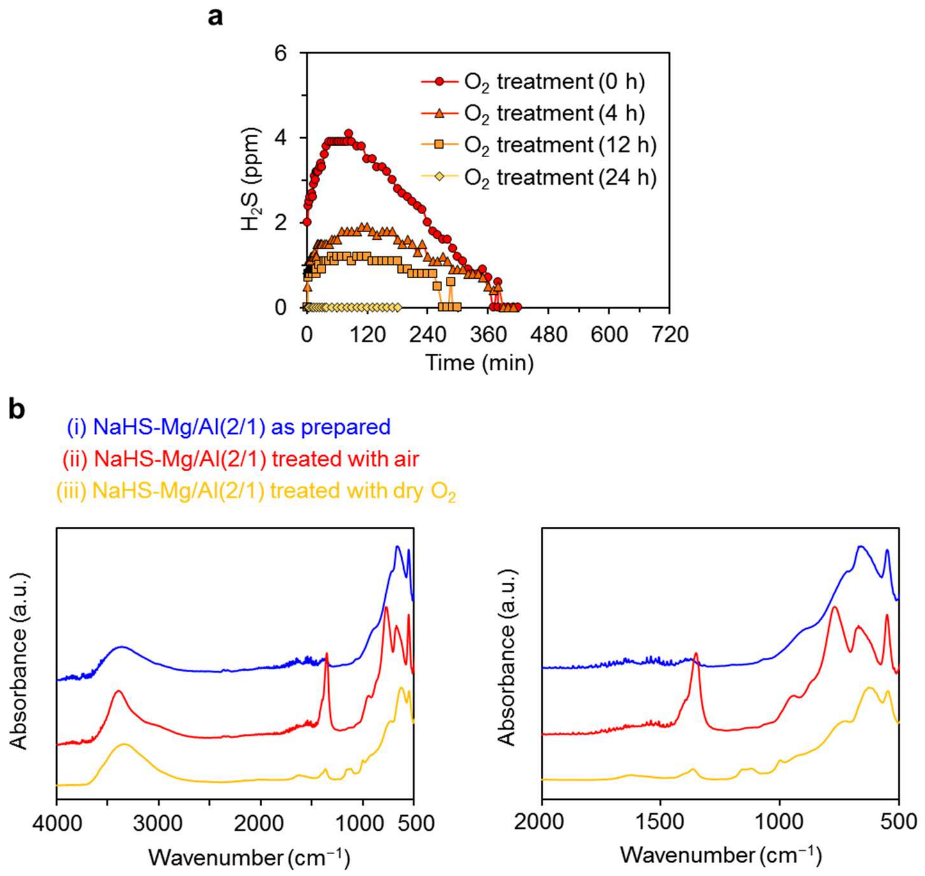

Supplementary Fig. 14. Influence of O_2. (**a**) H_2S release profiles after treatment with pure O_2. Two patches of NaHS-Mg/Al(2/1) (1.1 mg × 2) sandwiched between the porous tapes were evaluated under the standard flow condition. (**b**) IR spectra of NaHS-Mg/Al(2/1) in the forms of (i) as-prepared, (ii) after treatment with air for 24 h, and (iii) after treatment with dry O_2 for 24 h.

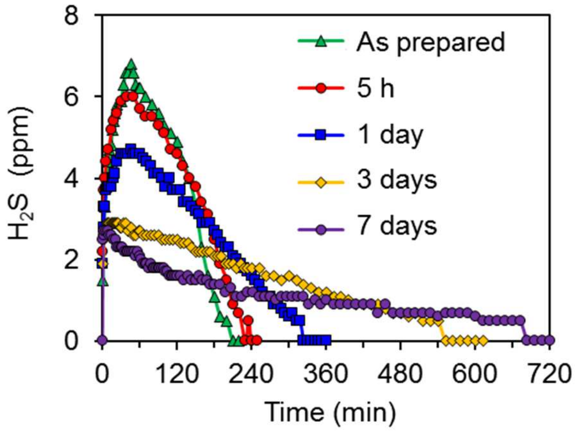

Supplementary Fig. 15. Heat treatment. Aging effect of NaHS-Mg/Al(2/1) under thermal treatment. Samples kept in gas barrier bag (Supplementary Fig. 9) were heated at 60 °C in an electric oven, whose interior was purged with dry N_2. Two patches of NaHS-Mg/Al(2/1) (1.1 mg × 2) sandwiched between porous tapes were evaluated under the standard flow condition.

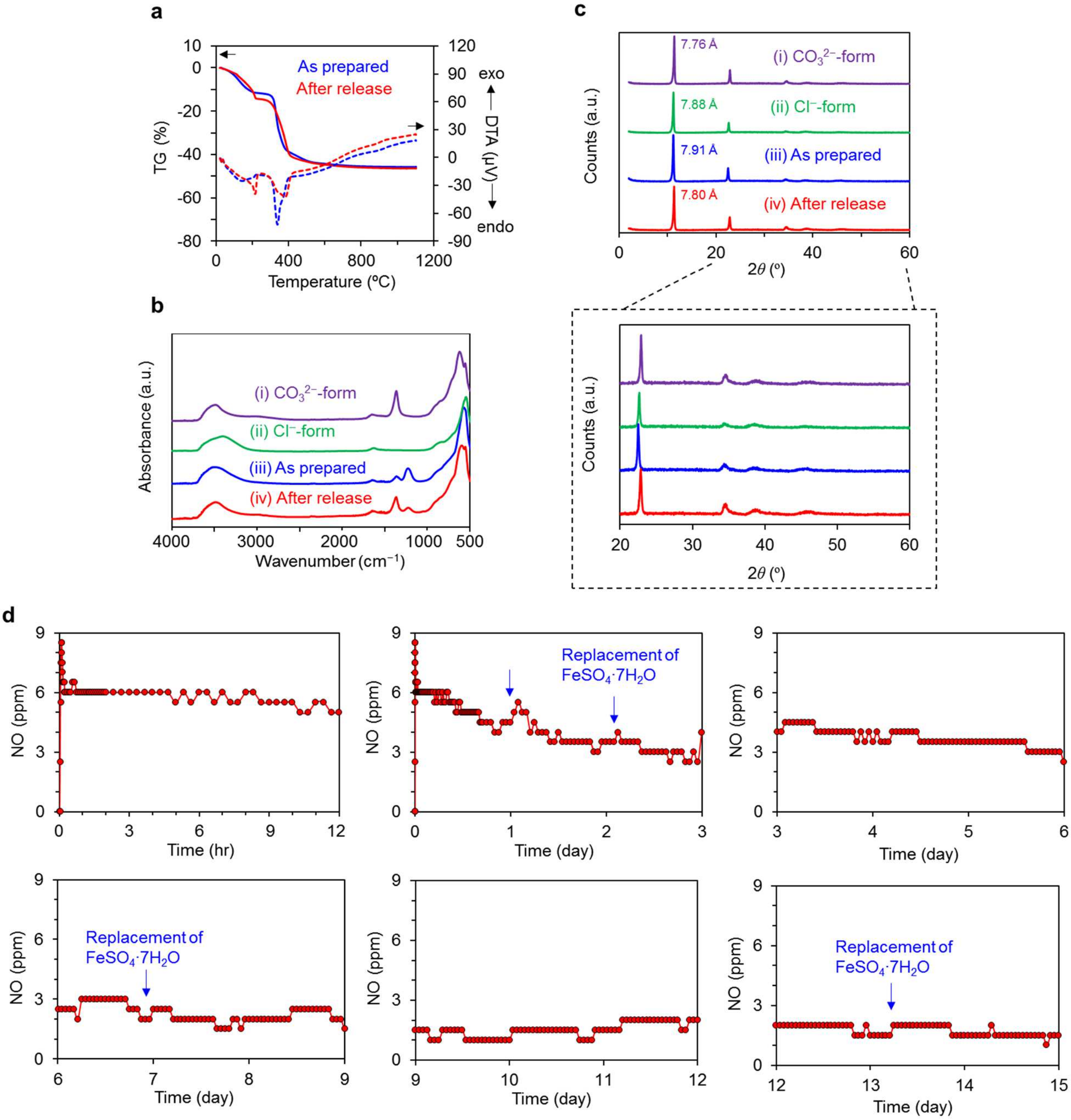

Supplementary Fig. 16. Characterization of HNO_2-releasing LDHs. TG-DTA profiles (**a**), IR spectra (**b**), and XRD patterns and basal spacing (**c**) of $NaNO_2$-Mg/Al(3/1) in the forms of as-prepared and after treatment with exhaled-breath for two weeks. For comparison, IR spectra and XRD patterns of CO_3^{2-}-Mg/Al(3/1) and Cl^--Mg/Al(3/1) are also shown. (**d**) Enlarged images of Fig. 4f.

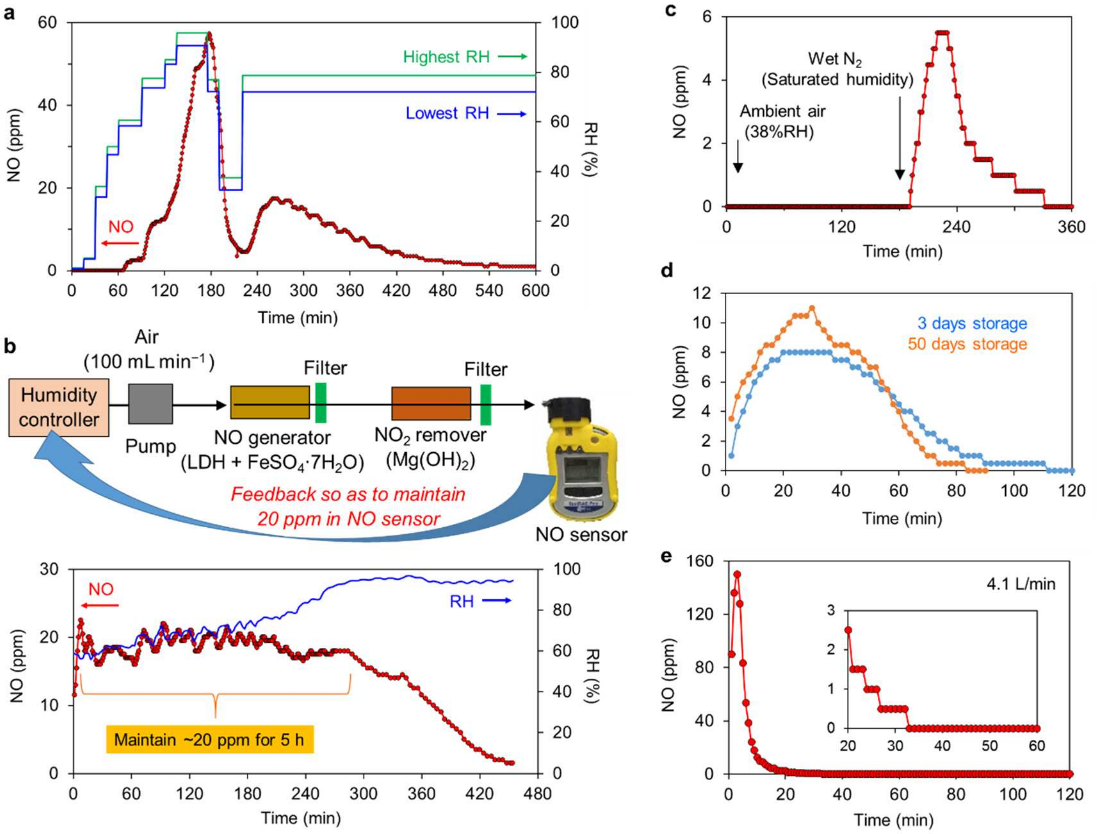

Supplementary Fig. 17. Detailed investigation of NO release. (**a**) Release of NO from $NaNO_2$-Mg(3/1) (20 mg) and $FeSO_4·7H_2O$ (0.2 g) mixture under air flow (100 mL min^{-1}) with variable humidity (0–95%RH). See Supplementary Fig. 6 for the experimental set-up. The NO concentration was lower than the limit of detection of the electrochemical sensor (<0.5 ppm) when the relative humidity of air was lower than 60%. In contrast, if RH was increased by more than 60%, NO release was initiated. Humidity was occasionally changed (shown as steps), and the highest and lowest RHs during each step are shown in the graph. (**b**) Manipulating NO concentration by combining NO sensor and humidity controller. Humidity of air delivered to NO generator was adjusted (operated manually) such that the NO sensor maintained a value of 20 ppm. Mixture of $NaNO_2$-Mg(3/1) (20 mg) and $FeSO_4·7H_2O$ (0.2 g) was used. (**c**) Release of NO from mixture of $NaNO_2$-Mg(3/1) (50 mg) and $FeSO_4·7H_2O$ (0.5 g). See Supplementary Fig. 5 for experimental set-up. The concentration of NO was lower than the limit of detection of the electrochemical sensor (<0.5 ppm) when ambient air (38%RH, 100 mL min^{-1}) was applied to the mixtures during the initial 3 h (note that NO was diluted with 4.0 L min^{-1} air). In contrast, the mixture started to release detectable amount of NO (>0.5 ppm) when wet N_2 (100 mL min^{-1}) was applied, indicating that humidity plays a critical role in NO generation. (**d**) Release of NO from $NaNO_2$-Mg(3/1) (50 mg) after 3 or 50 days storage. $NaNO_2$-Mg(3/1) was kept in a screw bottle (purged with dry N_2), and opened just before mixing with $FeSO_4·7H_2O$ (0.5 g). See Supplementary Fig. 5 for experimental set-up, where 100 mL min^{-1} ambient air (RH = 69%) and 4.0 L min^{-1} dry N_2 were utilized. (e) NO release from the mixture of $NaNO_2$ (20 mg) and $FeSO_4·7H_2O$ (200 mg). Experimental conditions are identical to Fig. 5a. This reaction caused a burst release of NO (About 6000 ppm in 100 mL min^{-1} flow) **[Caution!!]**. Particular attention should be paid for reaction scale.

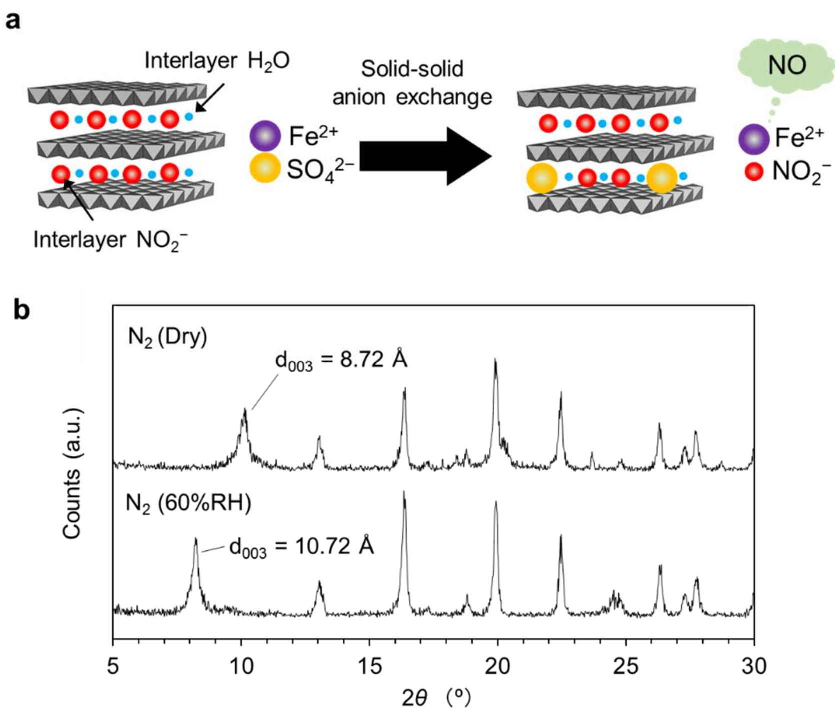

Supplementary Fig. 18. Mechanism of NO release from solid mixtures. (**a**) Proposed solid−solid state anion-exchange mechanism in $NaNO_2$-Mg(3/1) and $FeSO_4{\cdot}7H_2O$ mixture. Consequently, Fe^{2+} and NO^{2-} directly interacted (in other words, self-reactive $Fe^{II}(NO_2)_2$ was formed), leading to release of NO ($NO_2^- + Fe^{2+} + H_2O \rightarrow Fe^{3+} + 2OH^- + NO$). (**b**) XRD pattern of $NaNO_2$-Mg(3/1) and $FeSO_4{\cdot}7H_2O$ mixture after NO release, showing basal spacing (d_{003}) characteristic to SO_4^{2-}-type LDH (including response to humidity).

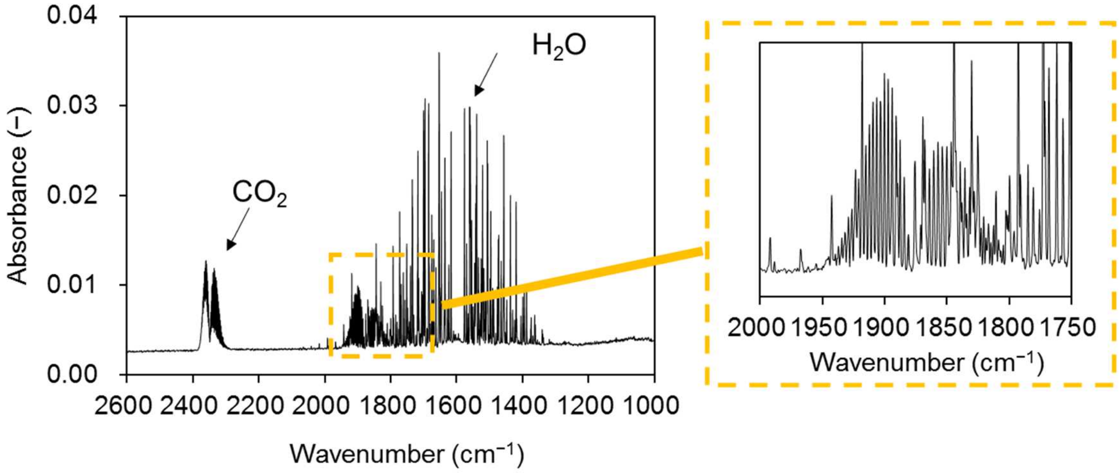

Supplementary Fig. 19. Characterization of NO released from LDH. IR spectrum of NO released from $NaNO_2$-Mg(3/1) (100 mg) and $FeSO_4{\cdot}7H_2O$ (1.0 g) mixture under wet N_2 (100 mL min^{-1}). NO was purified with $Mg(OH)_2$ (for removal of NO_2) and molecular sieve 3A (for removal of H_2O) before being injected into the gas cell (optical length = 10 cm, NaCl window). Note that neither N_2O (appear around 2200 cm^{-1}) nor NO_2 (appear around 1600 cm^{-1}) are observed in the spectrum. See Supplementary Fig. 7 for experimental details.

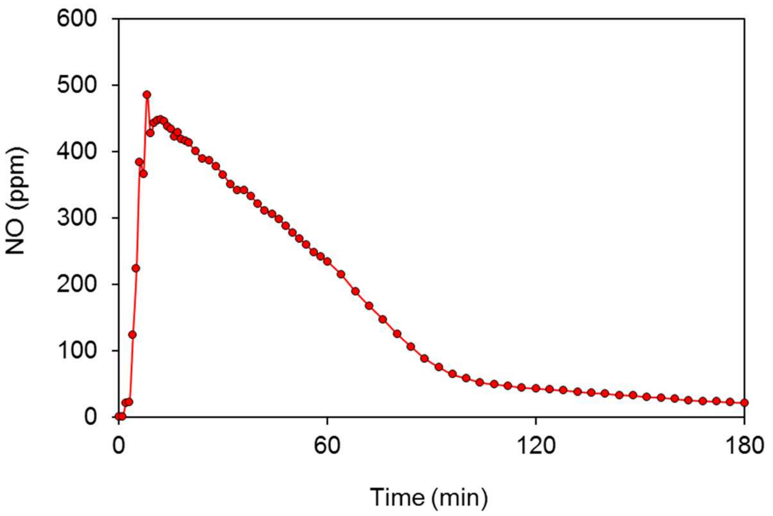

Supplementary Fig. 20. Characterization of NO released from LDH. Concentration of NO under N_2 flow (100 mL min^{-1}) monitored by chemiluminescent NO/NO_x analyzer. Mixture of $NaNO_2$-Mg(3/1) (100 mg) and $FeSO_4 \cdot 7H_2O$ (1.0 g) was used. See Supplementary Fig. 8 for experimental details.

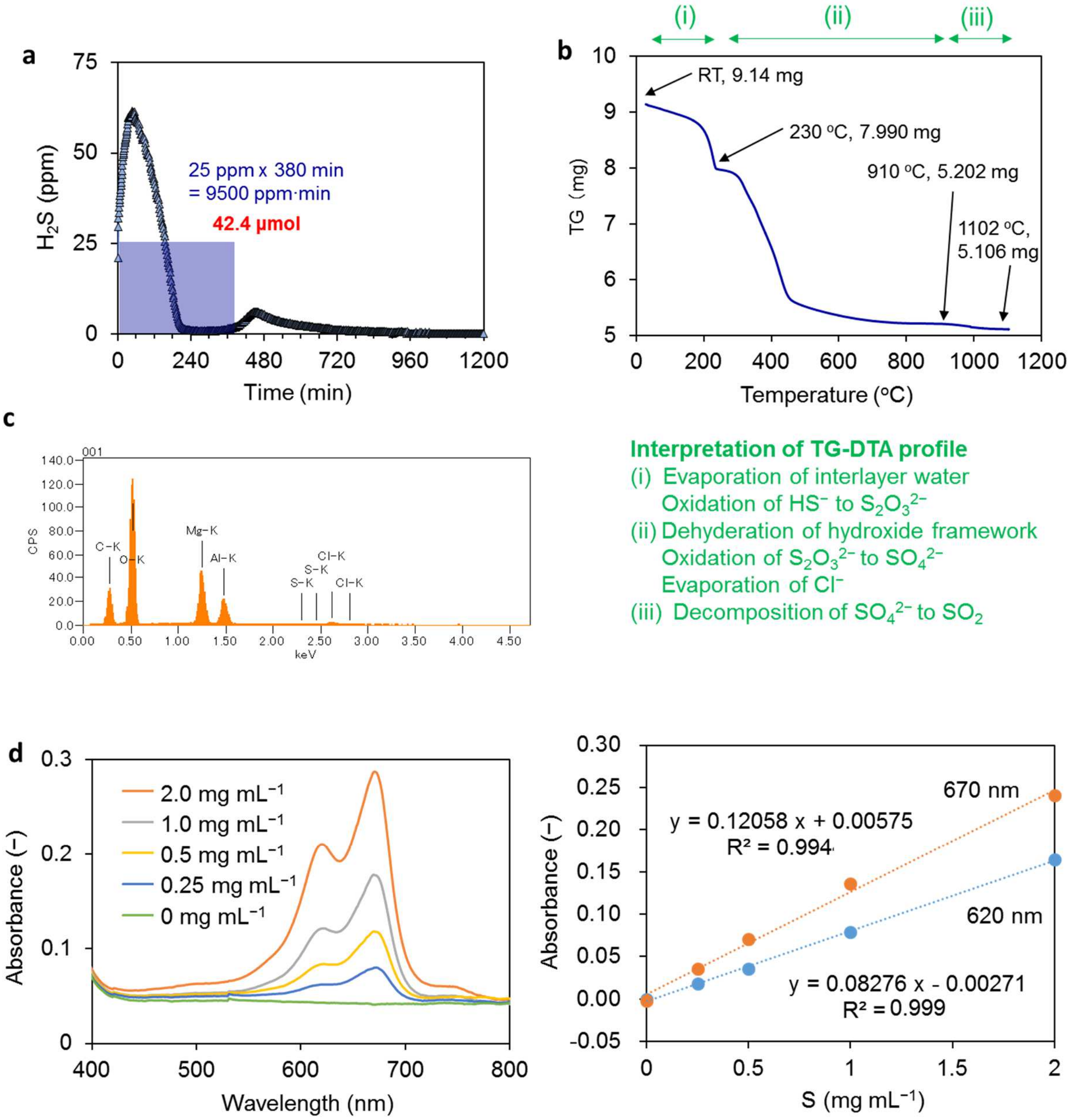

Supplementary Fig. 21. Quantification of HS^- involved in LDH. (**a**) H_2S release profile of 20 mg of powdery NaHS-Mg/Al(2/1) under the standard flow condition. (**b**) TG-DTA profile of NaHS-Mg/Al(2/1) after complete release of H_2S. Interpretation of the profile is also shown. (**c**) Typical EDS spectra of NaHS-Mg/Al(2/1) after release of H_2S. At least four EDS spectra were averaged to determine the atom ratio. (**d**) Calibration curve of sulfur (as HS^-/S^{2-}) in water based on methylene blue method. Standard aqueous solution of Na_2S (0, 0.25, 0.5, 1.0, and 2.0 mg mL^{-1}) were prepared by dissolving 35.45 mg of freshly opened anhydrous Na_2S (Dojindo Laboratories) in degassed deionized water (7.27 mL). The standard solution (5 μL) was added to 5.0 mL of degassed deionized water, and then analyzed by a test kit (WAK-S, Range = 0.1–5 mg L^{-1}, Kyoritsu Chemical-Check Lab., Corp.). UV-vis absorption spectra of the solution were measured at 25 °C using a quartz cell with 1 mm optical length.

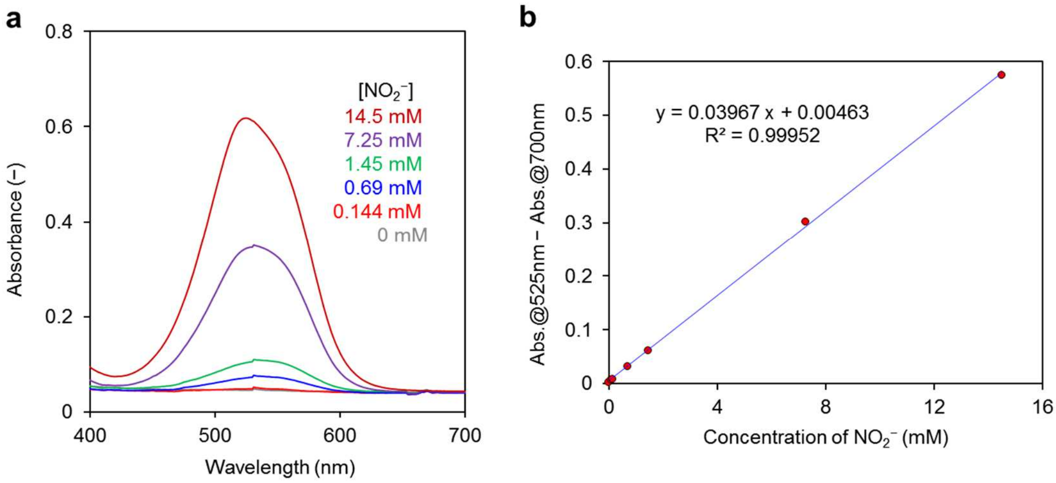

Supplementary Fig. 22. Quantification of NO_2^-. (**a**) UV-vis absorption spectra of Griess reagent responding to NO_2^-. 5 μL of $NaNO_2$ solution (0, 0.144, 0.69, 1.45, 7.25, and 14.5 mM) in water were added to 0.5 mL of aqueous solution of Griess reagent (40 mg mL^{-1}), wherein the Griess reagent was in large excess to NO_2^-. A quartz cell with 1 mm optical length was used for the measurement. (**b**) Calibration curve of NO_2^-, showing liner correlation between NO_2^- concentration and absorbance of Griess reagent.

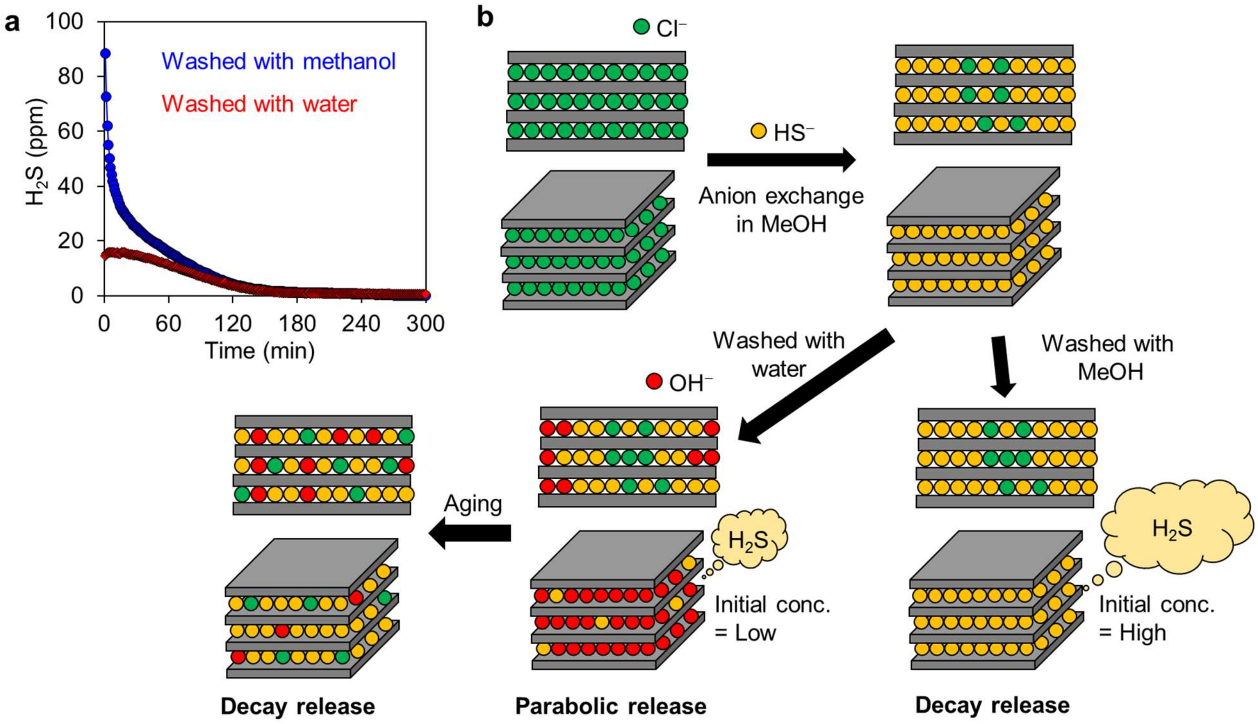

Supplementary Fig. 23. Mechanistic insight of gas release. (**a**) H_2S release profile (under the standard flow condition) of 5.0 mg of NaHS-Mg(2/1) prepared in MeOH. After completing two days reaction in MeOH, solid materials were filtrated on the membrane filter, and then washed with either methanol or water. (**b**) Plausible anion distribution model in LDHs for explaining their release profiles of H_2S and aging effect.

Supplementary Discussion

Quantification of HS^- involved in LDH.

The chemical formula of NaHS-Mg/Al(2/1) (prepared from 40 mg LDH and 36.6 mg $NaHS \cdot nH_2O$) was investigated by combing various analyses. First, H_2S release from 20 mg NaHS-Mg/Al(2/1) under standard flow condition (air, 50%RH, 100 mL min^{-1}, 20 °C) was monitored using electrochemical sensors, and the total amount of released H_2S was estimated to be 42.4 µmol based on the integration of the plot (Supplementary Fig. 21a). After complete release of H_2S (applying standard flow condition for 24 h), TG-DTA profile of NaHS-Mg/Al(2/1) was measured from RT to 1100 °C under air (Supplementary Fig. 21b). In addition, EDS spectra of NaHS-Mg/Al(2/1) before and after TG-DTA analysis were measured to determine the atom ratio of Mg, S, and Cl against Al (see Supplementary Fig. 21c for typical spectra). Thus, NaHS-Mg/Al(2/1) before TG-DTA analysis showed Mg/Al = 2.05, S/Al = 0.0386, Cl/Al = 0.158, and that after TG-DTA analysis showed Mg/Al = 2.06, S/Al = 0.0125, Cl/Al = not detected.

Taking TG-DTA profile, EDS, and charge-balancing into account, the chemical formula of calcined NaHS-Mg/Al(2/1) at 1102 °C was estimated as $Mg_{2.05}AlO_{3.54}(SO_4)_{0.0125}$. Besides, the tentative chemical formula of NaHS-Mg/Al(2/1) at 230 °C was set as $Mg_{2.05}Al(OH)_{6.1}(S_2O_3)_{0.0193}(Cl)_{0.158}(CO_3)_x$, where the amounts of Mg, S_2O_3, and Cl were determined based on the EDS result. The amount of (OH) was determined to maintain the charge balance of the layer (i.e., $Mg_{2.05}Al(OH)_{6.1}$) as +1. Based on the difference of mass in the TG-DTA profile of $Mg_{2.05}AlO_{3.54}(SO_4)_{0.0125}$ (1102 °C, 5.106 mg) and $Mg_{2.05}Al(OH)_{6.1}(S_2O_3)_{0.0193}(Cl)_{0.158}(CO_3)_x$ (230 °C, 7.990 mg), the amount of carbonate was determined as $x = 0.372$. Then, based on the difference of mass in the TG-DTA profile of $Mg_{2.05}Al(OH)_{6.1}(S_2O_3)_{0.0193}(Cl)_{0.158}(CO_3)_{0.372}$ (230 °C, 7.990 mg) and $Mg_{2.05}Al(OH)_{6.1}(HS)_{0.0386}(Cl)_{0.158}(CO_3)_{0.372} \cdot yH_2O$ (RT, 9.14 mg), the amount of hydrated water was determined as $y = 1.73$. Thus, the chemical formula of NaHS-Mg/Al(2/1) after H_2S release under air was confirmed.

Tentative chemical formula of NaHS-Mg/Al(2/1) before H_2S release was set as $Mg_{2.05}Al(OH)_{6.1}(HS_{non\text{-}emissive})_{0.0386}(HS_{emissive})_z(Cl)_{0.158}(OH)_w \cdot 1.73H_2O$. It was assumed that emissive $(HS_{emissive})_z$ and $(OH)_w$ will be replaced with $(CO_3)_{0.372}$ after exposure to air; thus, $(z + w)/2 = 0.372$. Then, the value of z was determined, such that 20 mg of NaHS-Mg/Al(2/1) released 42.4 µmol of H_2S. Consequently, the chemical formula of NaHS-Mg/Al(2/1) before release of H_2S was determined as $Mg_{2.05}Al(OH)_{6.1}(HS_{non\text{-}emissive})_{0.0386}(HS_{emissive})_{0.507}(Cl)_{0.158}(OH)_{0.237} \cdot 1.73H_2O$. Thus, majority (~93%) of HS^- involved in NaHS-Mg/Al(2/1) was released as H_2S, and ~7% was non-emissive due to aerial oxidation to $S_2O_3^{2-}$ and/or polysulfide. In the given chemical formula, 1 mg of NaHS-Mg/Al(2/1) contains 0.073 mg sulfur (S) source as an atom.

As an alternative approach, the amount of HS^- involved in NaHS-Mg/Al(2/1) was directly determined by the methylene blue method. In the globe box (dry N_2), 10 mg of NaHS-Mg/Al(2/1) was dispersed in 10 mL of degassed deionized water containing 60 mg of Na_2CO_3 (excess to HS^-). The suspension was sonicated for 2 min to disperse LDH, and the anion-exchange reaction between HS^- and CO_3^{2-} was promoted (LDH is known to show high affinity to CO_3^{2-}). Within 6 or 24 h of the anion exchange, ~10 mg (1 or 2 drops) of the suspension (the weight was exactly measured by balance) was added to 1.5 mL of degassed deionized water.

Then, the concentration of sulfur (as HS^{-}/S^{2-}) in the solution was analyzed by the methylene blue method using a commercially available test kit (WAK-S, Kyoritsu Chemical-Check Lab., Corp.). The UV-vis absorption spectrum of the blue solution was measured at 25 °C using a 1 mm quartz cell, and the concentrations of sulfur (as HS^{-}/S^{2-}) involved in the suspension after 6 h and 24 h anion-exchange reaction were estimated to be 0.069 mg mL^{-1} and 0.072 mg mL^{-1}, respectively, based on the calibration curve prepared separately (Supplementary Fig. 21d). This result indicates that ~0.07 mg of sulfur atom (as HS^{-}/S^{2-}) was released from 1 mg of NaHS-Mg/Al(2/1), which agrees with the aforementioned chemical formula of NaHS-Mg/Al(2/1) (i.e., 0.073 mg sulfur atom is involved in 1 mg NaHS-Mg/Al(2/1)).

Quantification of NO_2^{-} involved in LDH.

The amount of NO_2^{-} involved in $NaNO_2$-Mg/Al(3/1) was quantified using the Griess reagent. We dispersed 10.2 mg of $NaNO_2$-Mg/Al(3/1) in 10 mL of degassed deionized water containing 30 mg of Na_2CO_3. The suspension was sonicated for 1 min to disperse LDH, and left for 30 min to promote anion exchange between NO_2^{-} and CO_3^{2-} (LDH is known to show high affinity to CO_3^{2-}). The suspension (1 mL) was centrifuged to precipitate LDH, and then, 5 μL of the supernatant solution was added to 0.5 mL of the aqueous solution of Griess reagent (40 mg mL^{-1}).

The UV-vis absorption spectrum of the stained solution was measured at 25 °C using 1 mm quartz cell, and the NO_2^{-} concentration involved in the supernatant solution was estimated to be 2.74 mM based on the calibration curve prepared separately (Supplementary Fig. 22). This result indicates that 1.26 mg of NO_2^{-} was released from 10.2 mg of $NaNO_2$-Mg/Al(3/1), and the weight fraction of NO_2^{-} in $NaNO_2$-Mg/Al(3/1) was 12.4 wt.%. Note that the use of 60 mg of Na_2CO_3 for anion exchange yielded the same result. In addition, it was confirmed that the anion-exchange reaction was completed in 30 min as the same result was obtained after 2 h of the anion-exchange reaction.

Besides, SEM-EDS analysis showed that Al:Cl ratio in $NaNO_2$-Mg/Al(3/1) was approximately 1:0.09. Assuming that the molecular formula of $NaNO_2$-Mg/Al(3/1) was $Mg_3Al(OH)_8(Cl^{-}_{0.09}, NO_2^{-}{}_{0.91})\cdot 2H_2O$, according to the general formula of LDHs with neutral charge balance, the weight fraction of NO_2^{-} in the formula was 13.2 wt.%. The weigh fraction of NO_2^{-} determined by the Griess reagent method (i.e., 12.4 wt.%) was close to this value, and thus, the chemical formula of $NaNO_2$-Mg/Al(3/1) is close to $Mg_3Al(OH)_8(Cl^{-}_{0.09}, NO_2^{-}{}_{0.91})\cdot 2H_2O$.

It is estimated that 100 mg of $Mg_3Al(OH)_8(Cl^{-}_{0.09}, NO_2^{-}{}_{0.91})\cdot 2H_2O$ contains 0.287 mmol of NO_2^{-}, which corresponds to the release of 357 ppm NO for 180 min under 100 mL min^{-1} flow. The actual amount of NO released from LDHs was about half of the expected value (e.g., see Supplementary Fig. 20).

Additional discussion on H_2S release profile.

The H_2S release profiles of NaHS-Mg/Al(2/1) under air form a parabolic curve (e.g., Fig. 2i), where the H_2S concentration gradually increases and then decreases. If H_2S generation simply follows eq. 2 in Fig. 1b ($2[HS^{-}]_{LDH} + CO_2 + H_2O \rightarrow 2H_2S\uparrow + [CO_3^{2-}]_{LDH}$), the H_2S concentration solely depends on the amount of

$2[HS^-]_{LDH}$ (until the concentrations of CO_2 and H_2O are constant). Thus, the initial concentration of H_2S should be the highest and its release curve should be a simple decay type.

It was found that the use of methanol (MeOH) in the synthesis of NaHS-Mg/Al(2/1) (in reaction and washing processes) provided a decay curve in the release profile of H_2S with high initial concentration (Supplementary Fig. 23a). In contrast, if NaHS-Mg/Al(2/1) prepared in MeOH was washed with degassed deionized water several times, the initial concentration of H_2S was dramatically decreased. This result suggests that the interlayer HS^- located at the edge of layers was replaced with OH^- when washed with water (i.e., $[HS^-]_{LDH} + H_2O \rightarrow H_2S + [OH^-]_{LDH}$) (Supplementary Fig. 23b). As aerial CO_2 and H_2O also diffuse into the LDH interlayer from the edge, H_2S release will be low until CO_2 completes reaction with OH^- ($2[OH^-]_{LDH} + CO_2 \rightarrow H_2O + [CO_3^{2-}]_{LDH}$). Then, aerial CO_2 gradually reacts with HS^- located inside the LDH layer, resulting in a parabolic curve. The anion distribution model shown in Supplementary Fig. 23b also explains the aging effect of NaHS-Mg/Al(2/1) in H_2S release (e.g., Fig. 3i) as homogenization of the interlayer layer anion, recovering to a decay curve with reduced concentration.
