# Supplementary material for: Controlled release of H2S and NO gases through CO2-stimulated anion exchange
Source: Nat Commun. 2020 Jan 23;11:453. doi: 10.1038/s41467-019-14270-3 (PMC6978355; doi:10.1038/s41467-019-14270-3)
Supplement: Supplementary file 2 — Reporting summary [file 41467_2019_14270_MOESM2_ESM.pdf]

nature COMMUNICATIONS

MANUSCRIPT CHECKLIST

	Yes	N/A
**TITLE**		
No more than 15 words	✓	
Does not contain punctuation	✓	
**AUTHORS**		
Full postal address for all authors is provided	✓	
E-mail addresses provided for all corresponding authors	✓	
Maximum of 6 equally contributing authors (further contributions can be outlined in the author contribution statement)	✓	
Maximum of 6 joint supervisors (further contributions can be outlined in the author contribution statement)		✓
**ABSTRACT**		
No more than 150 words	✓	
Does not contain references	✓	
Results of the current study are written in present tense	✓	
Starts with short description of background (2-3 sentences)	✓	
Continues with presentation of the major results ('Here we show' or similar)	✓	
Ends with a description of the paper's conclusion	✓	
**MAIN TEXT**		
No more than 5,000 words in total (Introduction, Results, Discussion)	✓	
Section order is: Title, Abstract, Introduction, Results, Discussion, Methods, References, End Notes, Figure legends, Tables	✓	
Main text is provided as a Word or Tex document	✓	
Abbreviations are defined at first use	✓	
Genes and genotypes are italicized		✓
Mathematics: Scalar variables and constants should be italic, vectors should be bold without italics, subscripts and superscripts are displayed in non-italic font unless they are variables. Unit dimensions should be expressed using negative integers (e.g. kg m^{-1} s^{-2} not kg/ms^{2}) or the word 'per'	✓	
**Introduction**		
Less than 1,000 words	✓	
Contains no subheadings	✓	
Introduces the background and rationale for work	✓	
The last paragraph contains a brief summary of both the results and the conclusions (written in present tense)	✓	
Contains no reference to display items (unless overview figures are presented)	✓	
**Results**		
Divided by subheadings less than 60 characters (incl spaces) that do not contain punctuation	✓	
All data are shown either in the main text or the Supplementary Information	✓	
If personal communication from another laboratory is cited, written permission is provided		✓
Format of references to Supplementary items is (Supplementary Figure 1), (Supplementary Table 1), (Supplementary Note 1), (Supplementary Data 1), (Supplementary Movie 1)	✓	

nature
COMMUNICATIONS

MANUSCRIPT CHECKLIST

	Yes	N/A
**Discussion**		
Does not contain subheadings	✓	
Does not contain overlap with results section	✓	
**METHODS**		
Methods are contained within main paper wherever possible	✓	
Divided by subheadings less than 60 characters (incl spaces) that do not contain punctuation	✓	
Contain sufficient detail to repeat experiments (avoid 'as previously described')	✓	
Statement about availability of computer code, if important for main conclusions, is provided as a separate section under the heading "Code availability" after the data availability statement but before the References		✓
The paper conforms to our requirements on mandatory data deposition (see http://www.nature.com/authors/policies/availability.html)		✓
For all papers section "Data availability" is provided after the Methods section but before the References containing information on all mandatory and voluntary provision of research data. For further information refer to http://www.nature.com/authors/policies/data/data-availability-statements-data-citations.pdf	✓	
**REFERENCES**		
As a guideline, Articles allow up to 70 references	✓	
Numbered in the order they appear in the text, tables, figures and boxes	✓	
Formatted in Nature Communications style: 'Authors, Title, Journal, Volume, First-last page, (year)'	✓	
References to web-only journals include: 'Authors, Title, Journal, url/doi and year of publication		✓
References to websites include: 'Authors (if known), Title of page, url and year of publication		✓
References to preprint servers should be formatted as 'Authors. Preprint title. For example: Preprint at http://arxiv.org/abs/ YYMM.NNNN (Year)'		✓
Research datasets may be cited if they have been assigned digital object identifiers (DOIs) and should be formatted as 'Authors. Title. Publisher/repository, doi (year)'. Example: Smith, A. & Doe, J. Title. Figshare http://dx.doi.org/... (2016)	✓	
Contains only published work or work in press (including doi number)	✓	
Does not contain footnotes	✓	
**END NOTES**		
Acknowledgements are brief	✓	
Author contributions statement is provided	✓	
Conflict of interest statement is provided	✓	
**LEGENDS**		
Contain a brief title	✓	
No more than 350 words each	✓	
Every panel is described	✓	
Length of scale bars is defined	✓	
Definitions for new abbreviations/symbols/colours is provided	✓	
Error bars are defined as s.d. or s.e.m.		✓

nature COMMUNICATIONS

MANUSCRIPT CHECKLIST

	Yes	N/A
**DISPLAY ITEMS**		
No more than 10 total	✓	
Fit within a column/page (including legend)	✓	
Numbered in the order they appear in the main text	✓	
**Figures**		
Figures do not contain tables	✓	
Figure panels are arranged into a rectangular shape	✓	
Each panel is labelled with a single letter	✓	
Panels are not subdivided	✓	
Scale bars are included (but not labelled within the figure)	✓	
Blots and gels contain molecular weight or size markers		✓
Axes are labelled, including units	✓	
Stereo figures sufficient width apart (equivalent points separated by 5.5cm)		✓
Avoid the use of red and green in figures to avoid confusion for colour-blind readers (magenta and turquoise are alternatives)	✓	
**Tables**		
Include a title (no punctuation)	✓	
Tables are editable (not embedded as a picture in the document)	✓	
If table legend is required, it is displayed underneath the table		✓
NMR standard table for structural refinement statistics has been used (please see http://nature.com/authors/policies/tables-nmr.doc)		✓
X-ray standard table for structural refinement statistics has been used (please see http://nature.com/authors/policies/tables-xray.doc)		✓
Cryo-EM standard table for collection, refinement and validation statistics has been used (please see http://nature.com/authors/policies/tables-cryo-em.doc)		✓
**SUPPLEMENTARY INFORMATION**		
Provided as a single PDF file (except for Movies, Audio and Data)	✓	
Supplementary items are labelled and contain only these elements: Supplementary Figure 1 / Supplementary Table 1 / Supplementary Note 1 / Supplementary Discussion / Supplementary Methods / Supplementary References. Supplementary References appear at the end of the file.	✓	
Each Supplementary item is cited in the main text	✓	
Figure legends are displayed underneath each figure; ideally, each display item and its corresponding legend fit on one page	✓	
Format of the legends is the same as in the main manuscript and any error bars are defined (please see section above)	✓	
Movie legends are provided in the cover letter		✓
Supplementary References are numbered sequentially from 1 and are self-contained (they do not refer to the list of References in the main paper; any such papers are duplicated in the list of Supplementary References)	✓	
Where portions of blots and gels have been presented in the main paper, the full blots or gels are included in the Supplementary Information		✓
Supplementary Information does not contain essential display items (these should be displayed in the main text)	✓	
Supplementary Information does not contain Results	✓	
Supplementary Data files contain titles	✓	
Chemical structures are drawn using a Nature Chemistry Chemdraw template (please see https://www.nature.com/authors/guides/ChemStructureGuide.pdf and http://www.nature.com/authors/guides/NR_chemdraw_stylesheet.cds)		✓
